# Hemiboreal Stand Development and Living Biomass Carbon Stocks: An Ontogenetic and Typology-Based Assessment Using National Forest Inventory Data

**DOI:** 10.3390/plants15152344

**Published:** 2026-07-30

**Authors:** Raimundas Petrokas, Michael Manton, Gintaras Kulbokas, Milda Muraškienė

**Affiliations:** 1 Department of Forest Genetics and Tree Breeding, Institute of Forestry, Lithuanian Research Centre for Agriculture and Forestry, Liepų St. 1, LT-53101 Girionys, Lithuania; 2 Bioeconomy Research Institute, Vytautas Magnus University, Studentų St. 13, LT-53362 Akademija, Lithuania; 3 Lithuanian State Forest Service, Pramonės St. 11A, LT-51327 Kaunas, Lithuania; 4 Department of Silviculture and Ecology, Institute of Forestry, Lithuanian Research Centre for Agriculture and Forestry, Liepų St. 1, LT-53101 Girionys, Lithuania

**Keywords:** hemiboreal forests, forest development, ontogenetic strategies, forest typology, National Forest Inventory, growing stock, biomass turnover, carbon dynamics, closer-to-nature forest management

## Abstract

Carbon stocks in hemiboreal forests are frequently assessed using dominant-species or growing stock metrics, which may obscure ontogenetic and demographic processes governing biomass turnover. This study applied a process-based framework integrating succession theory, forest typology, and ontogenetic strategies to Lithuanian National Forest Inventory data reorganized by forest type-series. Three research expectations were evaluated: whether typology-based classification reveals patterns not visible under dominant-species reporting (H0), whether stand composition converges toward phase-typical configurations (H1), and whether fertile four-phase forests exhibit higher productivity and earlier CO_2_ flux reversals than three-phase forests (H2). H0 was rejected: forest type-series reorganization revealed contrasts in species assembly, stand structure, and carbon dynamics not detectable under dominant-species classification. H1 was supported: three-phase forests converged toward colonizer-dominated assemblies, while four-phase forests sustained multi-species, multi-cohort canopies with persistent broadleaved contributions. H2 was supported: four-phase forests exhibited higher gross volume increment and transitioned to negative CO_2_ accumulation 40 years earlier (61–80 years) than three-phase forests (101–120 years). Across both pathways, growing stock volume increased while CO_2_ accumulation turned negative in older stands, demonstrating divergence between cumulative biomass stock and net annual carbon flux. Forest Inventory data reorganized by forest type-series functioned as a process-based diagnostic tool supporting climate- and biodiversity-oriented management.

## 1. Introduction

Recent policy frameworks, including the EU Biodiversity Strategy for 2030 and the Forest Strategy for 2030 [1,2], call for management approaches that maintain forest ecosystem integrity under dynamic and increasingly uncertain conditions. Because forest degradation can occur even when canopy cover remains intact [3], assessments of ecosystem integrity must move beyond maximum sustained yield or simple productivity metrics and instead adopt indicators that evaluate ecological functioning relative to site-specific conditions. This shift aligns with the broader momentum toward EU policy such as the development and implementation of closer-to-nature forest management, which emulates natural disturbance regimes while fostering multifunctional forest landscapes [4,5,6]. As Angelstam et al. [7] emphasize, European forestry systems reflect diverse social-ecological contexts, yet all regions increasingly require management approaches that combine biodiversity conservation and timber production within a unified landscape planning framework.

The EU Nature Restoration Law (NRL) strengthens the need for habitat-level assessment tools capable of capturing ecosystem functions, structural diversity, and characteristic species assemblages [5]. The ontogenetic strategy model [8] effectively characterizes how plant communities respond to disturbance and site-specific gradients. Ontogenetic strategies therefore offer early-warning signals of regeneration shifts, compositional deviation, and emerging degradation that often precede measurable changes in canopy structure. Integrating ontogenetic strategy-derived vegetation data, particularly from the field layer, into national reporting could substantially improve the capacity to assess ecological condition, evaluate restoration outcomes, and demonstrate compliance with NRL targets related to habitat quality, species diversity, and ecosystem resilience.

Within this evolving policy landscape, National Forest Inventories (NFIs) can serve as critical enabling infrastructures for evidence-based forest governance [9]. Recent synthesis-oriented analyses of Lithuanian forest resources further demonstrate both the strengths of NFI-based monitoring and the challenges of interpreting large-scale forest dynamics based solely on aggregated statistical indicators [10]. By providing long-term, standardized measurements of forest structure, biomass, and landscape configuration, NFIs establish the empirical baseline necessary to detect deviations from natural reference conditions or policy-defined targets. When integrated with ecological integrity and resilience frameworks [11], these data allow the quantification of departures from natural forest dynamics, the assessment of historical management legacies, and the identification of stands and landscapes requiring restoration or adaptive intervention. NFIs thus form a crucial bridge between strategic policy ambitions for closer-to-nature, multifunctional forest management and the operational metrics needed for implementation and monitoring.

Naturally regenerating forests lie along a successional continuum describing development following disturbance rather than a simple recovery to a prior state toward site-adapted conditions [12,13]. At the advanced end of this continuum, old-growth stands express multi-layered structure, large trees, standing and downed deadwood, and horizontal heterogeneity—attributes reflecting long-term ecological processes [14]. Many key ecological processes, including regeneration trajectories, competitive hierarchies, and nutrient cycling, are mediated by understory vegetation and field-layer assemblages providing complementary indicators of developmental position [15,16]. These communities integrate site-specific conditions, disturbance history, and soil-microclimatic interactions, making them sensitive indicators of successional transitions and emerging degradation [17,18]. Their omission from forest monitoring constrains the early detection of resilience loss in stands where canopy structure remains stable [19,20,21]. Understanding forest development under climate change therefore requires mechanistic indicators that capture regeneration strategies and successional dynamics [22]. In hemiboreal systems, these processes regulate long-term carbon sequestration, structural development, and adaptive capacity [11,23,24]. Integrating strategy-based vegetation indicators into national forest assessment frameworks could improve the detection of ecological deviation, guide restoration prioritization, and strengthen the scientific foundation of closer-to-nature forest management.

Historically, European forestry has prioritized timber production through even-aged clearcutting systems aimed at sustaining high wood yield [25]. However, recent research has documented pronounced declines in forest growth across the hemiboreal region of Europe, reversing nearly a century of steady increase [26,27,28]. At the same time, production-oriented management has simplified forest structure, reduced species diversity, and homogenized age distributions (typically 0–100 years [29]), by maintaining continuous regeneration cycles and high volumes of marketable trees [30]. This trend is further reinforced by the scarcity of old-growth forests [31,32] and the diminished role of natural disturbance processes in shaping forest development [29,33,34].

A key limitation of contemporary forest monitoring arises from the definitions and accounting frameworks used to evaluate forest condition. International reporting systems, including FAO forest definitions and UNFCCC/IPCC greenhouse gas accounting frameworks, primarily characterize forests based on structural thresholds such as canopy cover, land use, and above-ground biomass [3,35]. While these metrics are effective for tracking forest extent and biomass stocks, they do not necessarily capture changes in structural complexity, species composition, or demographic processes. As a result, forests undergoing degradation through selective harvesting, structural simplification, or regeneration failure may still be classified as intact if canopy cover is maintained. This issue is explicitly recognized by the IPCC, which notes that direct human-induced forest degradation, particularly under continuous cover forestry systems, may remain undetected when assessments rely solely on canopy-based or aggregate biomass indicators [3].

These limitations have important implications for both carbon accounting and biodiversity assessment. Carbon stock estimates derived from growing stock or biomass provide an essential indicator of ecosystem functioning, particularly in relation to productivity, turnover, and disturbance dynamics. However, high carbon stocks do not necessarily imply high biodiversity, and in some cases, structurally homogeneous, even-aged stands may store substantial biomass while supporting limited species diversity. Conversely, forests managed under closer-to-nature or continuous cover approaches may exhibit lower standing biomass but higher structural heterogeneity and biodiversity. Therefore, carbon stock assessment should not be interpreted as a proxy for biodiversity, but rather as a complementary indicator that must be evaluated alongside structural and compositional metrics.

Taken together, these gaps highlight the need for integrated assessment frameworks that link carbon dynamics with forest structure, species composition, and demographic processes. Without such integration, current monitoring systems risk underestimating the extent of forest degradation and misinterpreting the ecological condition of forests, particularly in cases where canopy cover is maintained but long-term structural and functional decline occurs.

Addressing these limitations requires a process-based analytical framework that integrates carbon dynamics with forest structure, species composition, and successional dynamics across ecological gradients. Therefore, the overarching aim of this study is to develop a closer-to-nature, process-based assessment framework that links succession theory with ontogenetic strategy classification to assess forest development trajectories and carbon dynamics in hemiboreal forests. By integrating Lithuanian National Forest Inventory (LNFI) age-structured data with ontogenetic strategy classifications applied to both overstory tree species and field-layer vegetation, the study contrasts the limitations of dominant-species forest reporting [36] with an ecologically grounded, trait- and typology-based approach capable of detecting early structural and compositional shifts. To achieve this aim, the study pursues three objectives: (i) define stand-level successional phases using ontogenetic strategies assigned to tree species and field-layer communities, characterizing adaptive trade-offs and regeneration pathways across site conditions; (ii) examine age-structured growing stock composition across colonizer–successor categories of both forest types and forest type-series, comparing observed structures with successional phase-based reference profiles; and (iii) quantify live-tree carbon stocks and CO_2_-balance patterns across forest categories to evaluate how carbon accumulation and turnover differ between three-phase and four-phase trajectories, demonstrating how NFI-based metrics can strengthen biodiversity- and climate-oriented reporting frameworks.

Based on the analytical framework and variables defined above, the study formulates hypotheses aligned with each objective. Given the cross-sectional, space-for-time design of the study and the absence of formal inferential testing, these hypotheses are interpreted as structured research expectations guiding pattern identification and comparison across typological and ontogenetic gradients. Accordingly, results are presented in direct correspondence to these expectations across structural, compositional, and carbon-dynamic indicators.

**H1:**  *Successional Convergence Hypothesis (Objective i + ii): When forest stands are classified using ontogenetic strategies and forest type-series, age-structured patterns of species composition, growing stock distribution, and structural metrics (e.g., density, volume, mortality) will increasingly correspond to successional phase-typical configurations, as defined by LNFI-based reference profiles.*

**H2:**  *Productivity and Carbon Trajectory Hypothesis (Objective iii): Forests on fertile sites (four-phase trajectories) will exhibit (i) higher early–mid-age productivity (growing stock increment and density) and (ii) earlier transitions to negative net CO_2_ balance in living biomass, whereas nutrient-poor forests (three-phase trajectories) will show lower productivity but prolonged positive carbon accumulation across age classes.*

**H0:**  *Null Hypothesis: Structural, compositional, productivity, and living biomass carbon stocks do not differ systematically when stands are classified by forest type-series and ontogenetic strategies, and observed variation is fully explained by canopy-dominant species.*

## 2. Results

### 2.1. Volume Growth-Oriented Forest Inventory (Management Reporting Perspective)

**Figure 1:**
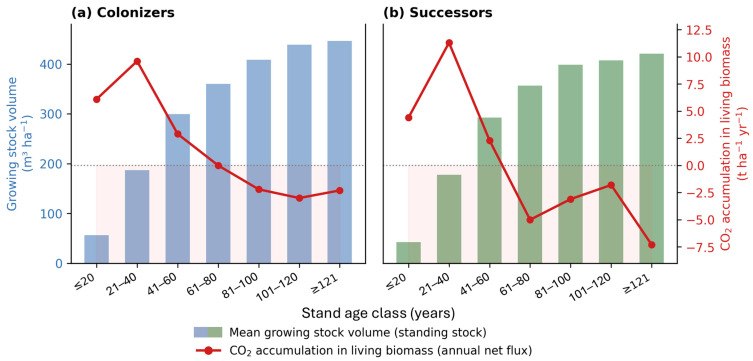
Mean growing stock volume (bars, left axis) and annual CO_2_ accumulation in living biomass (line, right axis) across stand-age classes for (**a**) colonizer and (**b**) successor forest types (LNFI data, Table 1). Standing volume continues to increase with stand age even as net annual carbon accumulation declines and becomes negative in older age classes, reflecting the decoupling of growing-stock increment from mortality-driven biomass turnover, including forest harvesting. The dashed horizontal line represents zero CO_2_ accumulation in living biomass (right axis), marking the threshold between net carbon sink (above) and net carbon source (below). The red shaded area indicates age classes where annual CO_2_ accumulation in living biomass is negative, meaning that mortality and harvesting removals exceed gross growth and the stand represents a net source of biomass carbon.

Results revealed that across both groups, growing stock volume increased monotonically with stand age while CO_2_ accumulation declined and turned negative in older age classes, with the reversal occurring earlier and more markedly in successor stands than in colonizer stands (Figure 1; Table 1).

Under the dominant-species (forest-type) classification framework, colonizer and successor stands collectively cover 2144 thousand ha of Lithuanian hemiboreal forests, with colonizer stands (1650.2 thousand ha) substantially outnumbering successor stands (493.8 thousand ha) (Table 1). Both groups display consistent age-dependent patterns in stand structure, productivity, and carbon dynamics, providing the baseline against which typology- and strategy-based analyses are evaluated in Section 2.2 and Section 2.3.

In colonizer stands, tree density declines progressively from 3017 trees ha^−1^ at ≤20 years to 824 trees ha^−1^ at ≥121 years, reflecting continuous self-thinning across the developmental gradient. Mean growing stock volume increases monotonically from 56.6 to 446.7 m^3^ ha^−1^ (Figure 1), while gross mean volume increment peaks at 11.5 m^3^ ha^−1^ yr^−1^ in the 21–40 year class before declining steadily to 7.1 m^3^ ha^−1^ yr^−1^ in the oldest stands. Tree mortality as a proportion of gross increment rises from 7.3% in the youngest stands to 38.4% at ≥121 years, marking a progressive shift from biomass accumulation to turnover-dominated stand dynamics. CO_2_ accumulation in living biomass peaks at 9.6 t ha^−1^ yr^−1^ at 21–40 years, declines to near-zero at 61–80 years (0.0 t ha^−1^ yr^−1^), and turns negative from 81–100 years onward (−2.2 to −3.0 t ha^−1^ yr^−1^), despite continued increases in growing stock volume, reflecting the decoupling between cumulative biomass stock and net annual carbon flux (Figure 1).

Successor stands exhibit a broadly similar structural trajectory but with notable differences in timing and magnitude. Tree density declines from 2521 to 662 trees ha^−1^ across the age gradient, and gross volume increment peaks at a higher value (12.3 m^3^ ha^−1^ yr^−1^ at 21–40 years) than in colonizer stands. However, tree mortality rises more sharply, reaching 48.2% of gross increment at ≥121 years, exceeding the equivalent colonizer value by nearly ten percentage points. As a result, CO_2_ accumulation peaks higher (11.3 t ha^−1^ yr^−1^ at 21–40 years) but declines more steeply, turning negative at 61–80 years (−5.0 t ha^−1^ yr^−1^) and reaching −7.3 t ha^−1^ yr^−1^ in the oldest age class (Figure 1). This more pronounced and earlier carbon flux reversal reflects intensified biomass turnover associated with higher mortality and structural reorganisation in successor-dominated stands.

### 2.2. Biodiversity- and Structure-Oriented Forest Inventory (Typology and Species-Assembly Perspective; H1)

Reorganization of the same inventory data by forest type-series revealed contrasting patterns of species assembly, stand structure, and successional composition between nutrient-poor three-phase and fertile four-phase forests. Age-structured patterns of species composition, stand density, growing stock volume, living biomass carbon stocks, and tree mortality were examined across forest type-series and ontogenetic strategy groupings.

#### 2.2.1. Three-Phase Forest Dynamics

Three-phase forests on nutrient-poor sites were strongly colonizer-dominated throughout all developmental stages (Table 2). Colonizer stands covered 629.3 thousand ha, substantially exceeding the 52.4 thousand ha of successor stands. In colonizer stands, tree density declined progressively from 2476 trees ha^−1^ at ≤20 years to 728 trees ha^−1^ at ≥121 years, while mean growing stock volume increased monotonically from 42.6 to 403.8 m^3^ ha^−1^. Gross volume increment peaked at 9.4 m^3^ ha^−1^ yr^−1^ at 21–40 years before declining to 6.3 m^3^ ha^−1^ yr^−1^ in the oldest stands, and tree mortality as a proportion of gross increment stabilized at 27–30% from mid-development onward. CO_2_ accumulation in colonizer stands peaked at 8.5 t ha^−1^ yr^−1^ at 21–40 years, declined through positive values to 0.8 t ha^−1^ yr^−1^ at 81–100 years, and turned negative from 101–120 years onward (−3.4 and −2.1 t ha^−1^ yr^−1^). Successor stands occupied a small total area and exhibited high variability across all indicators; their trends should be interpreted cautiously given the limited sample area. At the trajectory level, three-phase forests displayed structurally simple dynamics dominated by the persistence of early-established colonizer cohorts with low cohort turnover and limited successor recruitment.

#### 2.2.2. Four-Phase Forest Dynamics

Four-phase forests on fertile sites supported both colonizer and successor components across all developmental stages (Table 3). Colonizer stands covered 1020.9 thousand ha and successor stands 441.4 thousand ha. In colonizer stands, tree density declined from 3159 trees ha^−1^ at ≤20 years to 977 trees ha^−1^ at ≥121 years, while mean growing stock volume increased from 60.3 to 514.4 m^3^ ha^−1^—the highest recorded across all groups. Gross volume increment peaked at 12.3 m^3^ ha^−1^ yr^−1^ at 21–40 years before declining to 8.2 m^3^ ha^−1^ yr^−1^ in the oldest stands, and tree mortality as a proportion of gross increment rose from 7.6% at ≤20 years to 52.0% at ≥121 years. CO_2_ accumulation in colonizer stands peaked at 10.0 t ha^−1^ yr^−1^ at 21–40 years, turned negative at 61–80 years (−2.1 t ha^−1^ yr^−1^), and reached −5.5 t ha^−1^ yr^−1^ at 81–100 years. In successor stands, tree density declined from 2560 to 642 trees ha^−1^ and mean growing stock volume increased from 43.7 to 429.7 m^3^ ha^−1^. Gross volume increment peaked at 12.7 m^3^ ha^−1^ yr^−1^ at 21–40 years, the highest of any group, and tree mortality rose from 2.6% to 50.6% of gross increment at ≥121 years. CO_2_ accumulation in successor stands peaked at 11.9 t ha^−1^ yr^−1^ at 21–40 years before turning negative at 61–80 years (−6.0 t ha^−1^ yr^−1^) and reaching −6.7 t ha^−1^ yr^−1^ at ≥121 years. At the trajectory level, four-phase forests displayed higher productivity, greater structural complexity, and more intensified mortality dynamics across all age classes compared to the three-phase forests.

#### 2.2.3. Species Composition of Growing Stock

**Figure 2:**
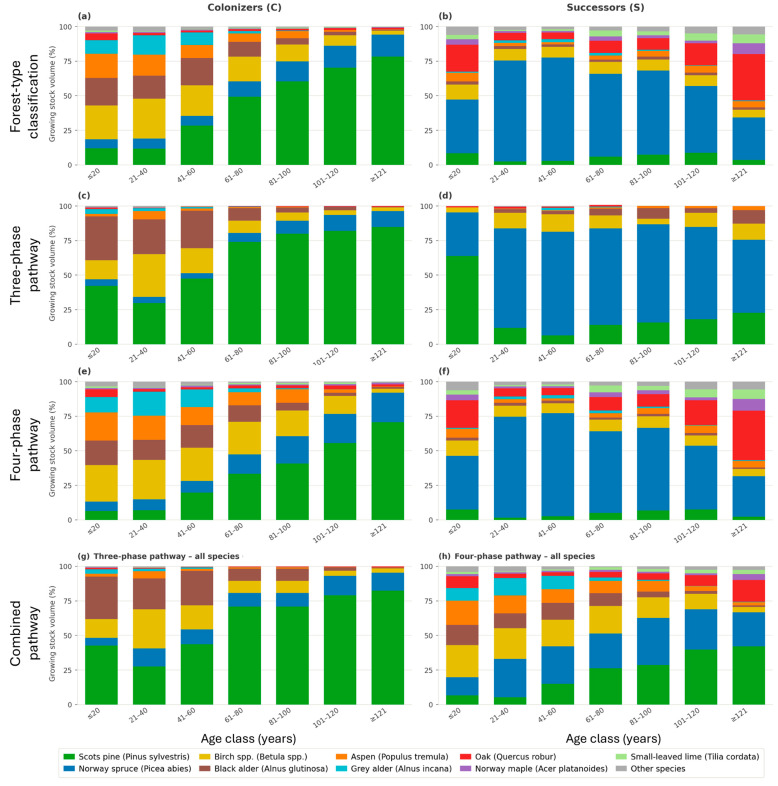
Species composition by growing stock volumes of the seven main tree species, (**a**) Colonizer forest types, (**b**) Successor forest types, (**c**) Colonizers in three-phase forests, (**d**) Successors in three-phase forests, (**e**) Colonizers in four-phase forests, (**f**) Successors in four-phase forests, (**g**) combined three-phase, and (**h**) The combined four-phase trajectory. Lithuanian National Forest Inventory age-structured growing-stock data were used to derive the data. Data can be found in Appendix A Table A1, Table A2 and Table A3. Percentages represent the dominant tree species contributing to growing stock volume.

Under forest-type grouping, colonizer forests were dominated by Scots pine (44% of mean growing stock volume), birch (18%), and black alder (12%), with pine-dominated stands covering 673.0 thousand ha of the total 1650.2 thousand ha (Figure 2a; Table 4; Table A1). Successor forests were dominated by Norway spruce (63%), oak (9%), and birch (8%), with spruce-dominated stands covering 399.0 thousand ha of the total 493.8 thousand ha (Figure 2b; Table 4; Table A1).

Reorganisation by forest type-series revealed contrasts not visible under forest-type reporting. Three-phase colonizer stands were strongly pine-dominated, with Scots pine accounting for 69% of mean growing stock volume, black alder for 11%, and birch for 10% (Figure 2c; Table 4; Table A2). Pine-dominated stands covered 435.5 of the total 629.3 thousand ha of three-phase colonizer area. Three-phase successor stands were dominated by Norway spruce (71%), with Scots pine contributing 13% and birch 10% (Figure 2d; Table 4; Table A2). Four-phase colonizer stands showed greater compositional diversity, with pine (28%), birch (23%), and spruce (13%) forming the three largest components of mean growing stock volume (Figure 2e; Table 4; Table A2). Four-phase successor stands were dominated by Norway spruce (62%), with oak contributing 10% and birch 8%, across a total area of 441.4 thousand ha (Figure 2f; Table 4; Table A2).

When all tree species within each pathway were considered together, the compositional trajectories of the three-phase and four-phase pathways diverged markedly from early development onward (Figure 2g–h; Table A3). In the three-phase pathway (Figure 2g; Table A3), early stands were co-dominated by Scots pine and black alder (42.8% and 30.9% respectively at ≤20 years), with birch contributing a secondary share (13.5%). Pine consolidated progressively with stand age, accounting for 70.9% of growing stock volume by 61–80 years and 82.4% in the oldest age class (≥121 years), while Norway spruce increased steadily to form the sole significant secondary component (13.0% at ≥121 years). Overall, the three-phase pathway mean was dominated by pine (66%), spruce (11%), and black alder (11%) (Table 4).

In the four-phase pathway (Figure 2h; Table A3), early stands displayed greater species richness, with birch (23.3%), aspen (17.6%), black alder (14.6%), Norway spruce (13.2%), grey alder (9.0%), and oak (8.5%) all contributing to growing stock at ≤20 years. Pine and spruce consolidated from mid-development onward, with pine reaching 39.7% and spruce 29.2% at 101–120 years. Oak increased in the oldest age class to 15.8% at ≥121 years, alongside pine (42.1%) and spruce (24.6%). The four-phase pathway mean was led by spruce (27%), pine (21%), and birch (18%) across all age classes (Table 4).

### 2.3. Climate-Oriented Forest Inventory (Carbon Dynamics and Turnover Perspective; H2)

**Figure 3:**
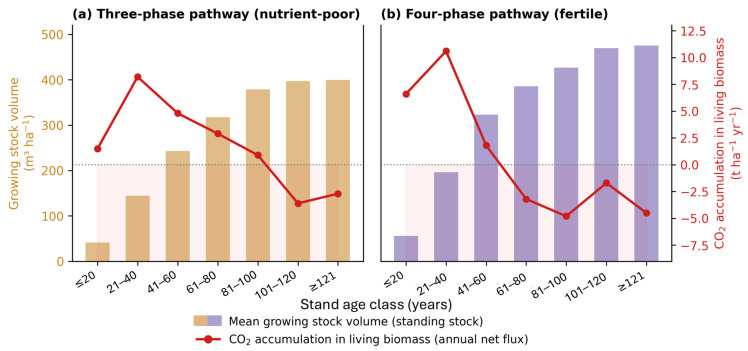
Growing stock volume and CO_2_ accumulation in living biomass across three-phase and four-phase forest pathways. Bars show mean growing stock volume (left axis) and the red line shows annual CO_2_ accumulation in living biomass (right axis) across stand-age classes for (**a**) the three-phase pathway on nutrient-poor sites and (**b**) the four-phase pathway on fertile sites (Table 2 and Table 3, “pathway” summary rows). Bars represent accumulated standing stock; the line represents the current annual net flux (growth minus mortality and harvesting removals) using the same D, BEF, R, and CF conversion parameters applied throughout. Four-phase forests reach higher standing volumes at every age class but transition into net carbon loss earlier (61–80 years) than three-phase forests, which maintain positive accumulation into late development (≥101–120 years), consistent with faster cohort turnover on fertile sites. The dashed horizontal line represents zero CO_2_ accumulation in living biomass (right axis), marking the threshold between net carbon sink (above) and net carbon source (below). The red shaded area indicates age classes where annual CO_2_ accumulation in living biomass is negative, meaning that mortality and harvesting removals exceed gross growth and the stand represents a net source of biomass carbon.

Carbon dynamics at the combined pathway level differed between three-phase and four-phase forest trajectories in the timing and magnitude of CO_2_ accumulation in living biomass (Table 2 and Table 3; Figure 3).

In the three-phase pathway, CO_2_ accumulation peaked at 8.2 t ha^−1^ yr^−1^ at 21–40 years, remained positive at 81–100 years (0.9 t ha^−1^ yr^−1^), and turned negative at 101–120 years (−3.6 t ha^−1^ yr^−1^), remaining negative at ≥121 years (−2.7 t ha^−1^ yr^−1^) (Figure 3a; Table 2). In the four-phase pathway, CO_2_ accumulation peaked higher at 10.6 t ha^−1^ yr^−1^ at 21–40 years but turned negative approximately 40 years earlier, at 61–80 years (−3.2 t ha^−1^ yr^−1^), reaching −4.8 t ha^−1^ yr^−1^ at 81–100 years (Figure 3b; Table 3). Across both pathways, mean growing stock volume increased monotonically from ≤20 years to ≥121 years while CO_2_ accumulation declined and turned negative in older age classes (Figure 3a–b; Table 2 and Table 3).

## 3. Discussion

### 3.1. Interpretation of Main Findings

This study evaluated three structured research expectations using the same LNFI dataset reorganized under two classification frameworks. The results support H1 and H2 and reject H0. Under dominant-species (forest-type) classification, colonizer and successor stands both exhibited consistent age-dependent declines in tree density, increases in growing stock volume, and progressive transitions from positive to negative CO_2_ accumulation in living biomass, patterns that provide a structural and productivity baseline but do not differentiate between ecological trajectories. When the same data were reorganized by forest type-series, contrasting developmental trajectories became apparent that were not detectable under dominant-species reporting, directly rejecting H0. In support of H1, age-structured patterns of species composition, stand density, growing stock volume, and tree mortality converged toward phase-typical configurations in both trajectory types: three-phase forests on nutrient-poor sites were strongly colonizer-dominated across all age classes, with Scots pine progressing from 42.8% at ≤20 years to 82.4% at ≥121 years and successor stands occupying only 52.4 thousand ha compared with 629.3 thousand ha of colonizer stands; four-phase forests on fertile sites sustained both colonizer and successor components across development, with broadleaved successors persisting in the oldest age classes and reaching 15.8% oak in growing stock at ≥121 years. In support of H2, four-phase forests exhibited higher early–mid-age gross volume increment (12.4 m^3^ ha^−1^ yr^−1^ at 21–40 years) compared with three-phase forests (9.4 m^3^ ha^−1^ yr^−1^) and transitioned to negative net CO_2_ accumulation approximately 40 years earlier, at 61–80 years (−3.2 t ha^−1^ yr^−1^), compared with 101–120 years (−3.6 t ha^−1^ yr^−1^) in three-phase forests. Across both pathways, growing stock volume increased monotonically with stand age while CO_2_ accumulation declined and turned negative in older age classes, demonstrating that net biomass flux and cumulative biomass stock follow divergent trajectories in later developmental stages.

The capacity of typology-based classification to reveal patterns not visible under dominant-species reporting is consistent with growing recognition in the literature that forest-type frameworks inadequately characterize ecological condition, particularly where canopy cover and aggregate biomass remain stable while successional dynamics, species assembly, and demographic processes diverge [3,29,35]. Mozgeris et al. [10] similarly noted that Lithuanian NFI data reliably documents increasing growing stock and expanding forest area at the national scale, but that aggregate trends of this kind remain difficult to interpret ecologically without process-based analytical frameworks. The contrasts in species assembly, structural complexity, and carbon dynamics identified here across three-phase and four-phase trajectories directly address this interpretive limitation.

The convergence of species composition and stand structure toward phase-typical configurations in both forest trajectory types aligns with established succession theory and with the ontogenetic strategy model [8,37]. In three-phase forests, the progressive consolidation of Scots pine from co-dominance with black alder in young stands to near-monoculture conditions at ≥121 years reflected the dominance of colonizer persistence strategies under nutrient-poor site conditions, where restricted successor recruitment and limited cohort diversification favored the long-term persistence of early-established colonizer cohorts [8,37]. The small total area of three-phase successor stands and their high variability across structural indicators further indicated restricted successor recruitment under these site conditions. In four-phase forests, the persistence of broadleaved successors across developmental stages and their re-emergence as a major component in the oldest age classes reflected the understory reinitiation and steady-state dynamics characteristic of four-phase trajectories on fertile sites [37,38,39,40], where gap-phase turnover and competitive filtering sustain multi-cohort, multi-species canopy structures over extended developmental timescales.

The earlier and more pronounced transition to negative CO_2_ accumulation in four-phase forests was consistent with the higher productivity and accelerated biomass turnover characteristic of more fertile sites. The positive relationship between site productivity and biomass turnover is well established in forest stand dynamics, where more productive sites support faster early growth but also accelerated competitive exclusion, mortality, and cohort replacement during later developmental stages [37,41,42]. This 40-year earlier transition to negative CO_2_ accumulation reflected this dynamic: the higher early gross volume increment of four-phase forests was followed by a more rapid rise in mortality as a proportion of increment, reaching 51.3% at ≥121 years compared with 27.0% in three-phase forests. By contrast, the more prolonged positive carbon accumulation in three-phase forests reflected the slower biomass turnover associated with colonizer-dominant, pine-dominated stands on nutrient-poor sites, where the longevity of Scots pine and lower competitive intensity delayed the transition from biomass accumulation to turnover-dominated dynamics [43].

The stabilization of carbon sequestration rates at the national scale reported by Mozgeris et al. [10] may partially reflect this transition from growth-dominated biomass accumulation to turnover-dominated biomass dynamics in ageing Lithuanian forests, a pattern that the typology-based framework applied here demonstrates occurs at systematically different developmental stages depending on forest trajectory and site productivity.

### 3.2. Implications for Forest Management and Carbon Accounting

This study has several direct implications for forest management and for the design of carbon accounting frameworks based on National Forest Inventory data, particularly in the context of national and European reporting obligations under the UNFCCC and the EU Forest Strategy for 2030 [1,2].

National and international reporting frameworks that rely primarily on biomass stock metrics may fail to capture transitions to net carbon source status that occur in older stands where mortality and harvesting removals exceed gross growth, particularly in four-phase forests, where this transition occurred at 61–80 years across both colonizer and successor components. These findings support the recommendation of Manton et al. [29] and others [3,35] that forest carbon reporting frameworks should incorporate flux-based indicators alongside stock-based metrics, enabling detection of demographic turnover-driven carbon losses that would otherwise remain hidden within aggregate growing stock increases.

The typology-based framework demonstrated here provides a practical tool for differentiating between persistence-dominated trajectories, characteristic of three-phase forests on nutrient-poor sites, and high-turnover trajectories, characteristic of four-phase forests on fertile sites. This differentiation has direct relevance for management decisions concerning harvest timing and intervention intensity. In four-phase colonizer stands, tree mortality reached 52.0% of gross volume increment at ≥121 years—the highest value recorded across all groups, indicating that biomass losses through natural mortality were comparable in magnitude to gross production. Under these conditions, forest management strategies that defer harvesting in expectation of continued net carbon accumulation may not achieve the intended carbon sequestration outcomes, as net living biomass carbon flux had already turned negative by 61–80 years. By contrast, three-phase colonizer stands maintained positive CO_2_ accumulation until 101–120 years and mortality stabilised at 27–30% of gross increment from mid-development onward, indicating a more stable carbon accumulation trajectory that supports longer rotation or retention periods without equivalent risk of net carbon source status.

Integrating forest type-series classification and ontogenetic strategy groupings into standard NFI reporting protocols would enable national inventories to provide process-based diagnostic indicators of forest developmental stage, successional trajectory, and carbon flux status—information directly relevant to assessing restoration progress, identifying stands at risk of ecological degradation, and evaluating the effectiveness of closer-to-nature management interventions [4,5,6,29]. This integration would also strengthen compliance with EU Biodiversity Strategy for 2030 and Nature Restoration Law obligations [1,5], which require assessment tools capable of capturing ecological condition beyond the canopy cover and aggregate biomass thresholds on which current monitoring frameworks primarily rely [3].

### 3.3. Methodological Considerations and Limitations

The interpretation of the results presented here is subject to several methodological and conceptual constraints that should be considered when evaluating the patterns reported.

The analysis was based on cross-sectional LNFI data in which stands of different chronological ages were compared as a proxy for successional development, following a space-for-time substitution approach. This method enabled large-scale assessment of structural and functional patterns across typological groupings but did not track the temporal evolution of individual stands. Consequently, differences observed among age classes may reflect not only successional dynamics but also spatial heterogeneity in site conditions, legacies of past management practices, historical disturbance regimes, variation in species composition at establishment, and climatic influences acting differentially across the landscape. The identified patterns should therefore be interpreted as large-scale, aggregated tendencies in forest development across typological and ontogenetic groupings rather than as deterministic representations of stand-level temporal trajectories.

A related conceptual limitation concerns the relationship between chronological age and ontogenetic developmental stage. Chronological stand age was used as a proxy for successional position throughout this study, consistent with standard NFI practice. However, stand age does not fully capture the complexity of successional development, particularly in stands affected by partial disturbance, structural reorganisation, or divergent regeneration histories. In three-phase forests, where the longevity of Scots pine can extend developmental timescales well beyond the age-class boundaries used here, chronological age may underestimate the actual ontogenetic position of some stands. In four-phase forests, where gap-phase dynamics and understory reinitiation generate multi-aged structural complexity within individual stands, a single chronological age class integrates stands at genuinely different ontogenetic stages. These factors may introduce heterogeneity within age classes that is not captured by the aggregated LNFI summaries used in this analysis.

The scope of carbon accounting in this study was restricted to living biomass pools, incorporating above-ground and below-ground biomass via standard biomass-expansion and root-to-shoot parameters. Other major forest carbon pools, including deadwood, forest floor litter, mineral soil organic carbon, and harvested wood products, were not included due to data limitations within the LNFI framework. Consequently, reported CO_2_ accumulation and loss values represent changes in living biomass carbon only and should not be interpreted as full ecosystem carbon balances. In older developmental stages, where deadwood accumulation and soil carbon dynamics may become increasingly significant components of total ecosystem carbon flux, this restriction may result in underestimation of the magnitude and complexity of ecosystem-level carbon dynamics.

Carbon estimates were derived from fixed biomass-conversion parameters applied uniformly across species groups, following IPCC Tier 1 guidance. While this approach ensured methodological consistency and comparability at the national scale, it did not capture species-specific variation in wood density, biomass allocation ratios, or carbon fractions. In mixed-species stands characteristic of four-phase forests, where colonizer and successor species with contrasting wood properties co-occur across developmental stages, this generalisation may introduce systematic bias in carbon stock and flux estimates for individual species groups. The reported values should therefore be interpreted as generalized approximations of living biomass carbon dynamics consistent with Tier 1 accounting approaches rather than as precise species-level or stand-level carbon budgets.

### 3.4. Link to Biodiversity and Restoration Objectives

The structural and compositional patterns identified in this study have direct implications for biodiversity assessment and restoration-oriented forest management, particularly in the context of the EU Nature Restoration Law and the Biodiversity Strategy for 2030 [1,5], which require habitat-level assessment tools capable of capturing structural diversity, characteristic species assemblages, and ecological functioning.

Three-phase forests on nutrient-poor sites converged toward structurally simplified, colonizer-dominated canopies with limited species diversity, low cohort turnover, and restricted successor recruitment, while four-phase forests on fertile sites maintained higher species richness, active successor recruitment, and increasing structural complexity through gap-phase dynamics and understorey reinitiation across the developmental gradient. These contrasting structural trajectories have direct consequences for habitat availability. The dominance by a single long-lived pioneer species, low structural heterogeneity, and absence of multi-cohort dynamics characteristic of three-phase forests are associated with reduced habitat availability for species dependent on structural diversity, canopy gaps, and late-successional microhabitats [14,19,21]. The re-emergence of oak and other broadleaved successors in the oldest four-phase stands is particularly relevant from a biodiversity perspective, as oak-associated habitats support disproportionately high species richness across multiple taxonomic groups in European temperate and hemiboreal forests [7,14,29].

The finding that high carbon stocks in living biomass did not correspond to high structural complexity or species diversity, most evident in older three-phase colonizer stands, where pine monocultures accumulated substantial growing stock while sustaining minimal successor recruitment, has direct implications for biodiversity-oriented forest management and assessment. Carbon stock metrics derived from growing stock volume systematically overestimate ecological condition in structurally simplified stands, while underrepresenting the biodiversity value of structurally complex stands with lower but more dynamically distributed biomass [3,29,31]. This distinction is relevant to the implementation of the Nature Restoration Law [5], where restoration targets require indicators of ecological condition that capture structural and compositional attributes beyond aggregate biomass thresholds. Ontogenetic strategy classification and forest type-series frameworks, as applied here, provide such indicators by characterising successional position, cohort structure, and species assembly in terms directly linked to habitat quality and biodiversity potential.

The integration of typology-based classification with ontogenetic strategy groupings into NFI reporting could substantially improve the capacity of national monitoring systems to detect structural degradation, evaluate restoration trajectories, and demonstrate compliance with EU biodiversity and restoration targets [1,2,5,9]. By linking standardised inventory variables, stand density, species composition, growing stock, and mortality, to successional phase-typical reference profiles, the framework proposed here enables NFI data to function as a process-based diagnostic tool for assessing both live biomass carbon stocks and biodiversity outcomes within a unified analytical structure.

## 4. Materials and Methods

### 4.1. Conceptual Background

#### 4.1.1. Stand Successional Phases

Two classical models describe compositional change along successional gradients: relay floristics, in which species replace one another sequentially, and initial floristics, where multiple species establish early but dominate at different times [37]. Competition, stress, and disturbance regulate these trajectories, driving increases in tree size, structural complexity, and species diversity [44]. The ontogenetic strategy model integrates these dynamics into a successional phase-based framework that provides a consistent basis for monitoring and interpreting forest change. In this framework, forest development is interpreted as a trajectory constrained by the interaction between contrasting ontogenetic strategies and site-specific ecological conditions.

Following disturbance, stands initially occupy a broad range of structural and species-compositional configurations that are gradually filtered by resource limitation, competitive hierarchies, and ontogenetic-strategy sorting—fundamental mechanisms in successional and trait-based ecology [37,45,46]. As these processes act over time, forests converge toward recurrent configurations characteristic of Lithuania’s forest type-series and the successional phases used in this study. Structural legacies such as deadwood, understory composition, and soil organic carbon further constrain these trajectories by retaining information about earlier ecological conditions [11,33].

Building on foundational work in stand dynamics [41,42], we define a four-phase forest trajectory consistent with patch dynamics theory [43,44,45] (Table 5). Stand initiation (1) follows a stand-replacing disturbance, such as clear-cutting, windthrows, or insect outbreak, that resets the developmental trajectory and provides high light availability at the forest floor [47,48]. Pioneer species, including Scots pine, birch, aspen, and alder, establish rapidly and form an even-aged canopy, typically persisting for 15–40 years [37,47].

Stem exclusion (2) begins with canopy closure, which reduces light availability and intensifies competition among overstory trees. Species richness and regeneration decline, and only shade-tolerant individuals persist in the understory [37]. The duration of this phase depends largely on the longevity of pioneer dominants; for example, aspen and birch typically persist for several decades, whereas Scots pine may dominate for more than two centuries [43].

Understory reinitiation (3) emerges as mortality among early dominants creates canopy gaps that permit suppressed individuals and late-successional species to be recruited [37]. This phase initiates structural diversification and the development of mixed-age cohorts.

The steady state phase (4) is reached when mortality and recruitment approximate dynamic equilibrium, resulting in multi-species, multi-cohort, vertically structured canopies, broad age distributions, and late-successional attributes such as large trees, deadwood, and spatial heterogeneity [29,44,49,50,51].

The age ranges assigned to each successional phase are approximate and partially overlapping by design. This reflects the continuous and process-based nature of forest development, where transitions between phases are gradual and influenced by site conditions, disturbance history, and species composition. The use of overlapping intervals therefore does not indicate discrete chronological boundaries but rather represents typical windows within which specific structural and functional processes dominate. In this context, chronological age serves as an indicative proxy for developmental stage, while recognizing that multiple successional processes may occur simultaneously within a given stand. The assignment of species-specific age ranges is based on a synthesis of (i) published stand-dynamics and succession literature [22,37,52,53], (ii) species life-history traits and longevity patterns, (iii) established Lithuanian forest typology and successional classifications [43,54], and (iv) consistency checks against age-structured patterns observed in LNFI datasets. Accordingly, the ranges should be interpreted as ecologically informed reference intervals rather than empirically derived thresholds for individual species.

**Table 5:** Conceptual framework of a four-phase forest developmental trajectory based on approximate age ranges. Age intervals are indicative and intentionally overlapping, reflecting the gradual and non-linear nature of successional transitions rather than discrete boundaries between phases. This framework synthesizes age-class distributions to illustrate stand development across the four successional phases [37,47,55,56].

Stand Initiation (~0–30) * Pioneers (Colonizers)	Stem Exclusion (~10–80) Pioneers (Colonizers)	Understory Reinitiation (~30–100) Post-Pioneers (Successors)	Steady State (~60–180)
*Pinus sylvestris* (0–30)	*Pinus sylvestris* (31–80)	*Quercus robur* (40–100)	*Pinus sylvestris* (81–140)
*Betula pubescens* (0–20)	*Betula pubescens* (21–50)	*Fraxinus excelsior* (40–90)	*Betula pendula* (61–110)
*Betula pendula* (0–20)	*Betula pendula* (21–60)	*Carpinus betulus* (30–70)	*Alnus glutinosa* (61–110)
*Salix fragilis* (0–20)	*Salix fragilis* (21–40)	*Picea abies* (30–70)	*Quercus robur* (101–180)
*Salix alba* (0–20)	*Salix alba* (21–60)	*Ulmus minor* (30–80)	*Fraxinus excelsior* (91–140)
*Populus tremula* (0–20)	*Populus tremula* (21–40)	*Ulmus glabra* (30–80)	*Carpinus betulus* (71–120)
*Alnus incana* (0–10)	*Alnus incana* (11–30)	*Ulmus laevis* (30–80)	*Picea abies* (71–120)
*Alnus glutinosa* (0–20)	*Alnus glutinosa* (21–60)	*Tilia cordata* (30–80)	*Ulmus minor* (81–130)
		*Acer platanoides* (30–70)	*Ulmus glabra* (81–130)
			*Ulmus laevis* (81–130)
			*Tilia cordata* (81–130)
			*Acer platanoides* (71–120)

* Note: Overlapping age ranges reflect gradual transitions between successional phases and should be interpreted as indicative rather than discrete thresholds.

#### 4.1.2. Ontogenetic Strategy Model

To interpret stand development and age-structured patterns in hemiboreal forests, this study applies the ontogenetic strategy model [8] together with succession theory. This integrated framework assumes that forest structural trajectories arise from adaptive trade-offs among tree species in response to light gradients, disturbance regimes, and resource limitation [57,58]. Accordingly, two functional groups, colonizers (C) and successors (S), represent contrasting ontogenetic strategies that shape regeneration pathways, competitive hierarchies, and long-term carbon dynamics.

Colonizers comprise pioneer species such as Scots pine (*Pinus sylvestris*), two birch species (*Betula pendula* and *B. pubescens*), Eurasian aspen (*Populus tremula*), and alder species (*Alnus incana* and *A. glutinosa*), which regenerate rapidly in open, high-light environments. Their traits emphasize fast biomass accumulation, early reproduction, and efficient dispersal, enabling dominance in stand initiation and stem-exclusion phases. In contrast, successors, including English oak (*Quercus robur*), European ash (*Fraxinus excelsior*), hornbeam (*Carpinus betulus*), Norway spruce (*Picea abies*), elm species (*Ulmus* spp.), small-leaved lime (*Tilia cordata*), and Norway maple (*Acer platanoides*), are adapted to shaded, resource-moderate to stable conditions, establishing in canopy gaps and forming multi-cohort, structurally complex late-successional stands. These strategies reflect species-specific adjustments of photosynthetic capacity, crown architecture, litter quality, and below-ground interactions, thereby influencing carbon inputs, turnover, and long-term soil carbon storage [59,60,61].

Linking age-structured growing stock patterns to ontogenetic strategies therefore supplies an analytical basis for evaluating how species contribute to forest structure and carbon cycling across successional phases. By embedding ontogenetic strategies within a typology-stratified sampling design, the methodology captures the dynamic equilibrium among regeneration pathways, site fertility, and structural turnover, thereby addressing known gaps in forest degradation accounting as highlighted by the IPCC [3] and supporting the development of closer-to-nature forest management and resilience-oriented forest reporting at national scale.

For operational consistency with the LNFI species-resolution framework, the four-strategy model of Petrokas et al. [8], which distinguishes gap colonizers, gap successors, gap fillers, and gap competitors, is operationally condensed here into two broad functional groups: colonizers (C) or pioneer species and successors (S) or post-pioneer species. The successor group therefore encompasses species with differing shade tolerance, growth rates, longevity, mortality dynamics, and biomass turnover characteristics, including Norway spruce, oak, ash, lime, and maple. This aggregation reflects the binary nature and resolution limits of the LNFI dataset, which does not provide sufficient trait-level or regeneration process information to robustly distinguish between finer ontogenetic sub-strategies at national scale. While analytically necessary, this simplification may mask important ecological differences among successor species. For example, coniferous species such as Norway spruce, exhibit distinct growth patterns, shade tolerance, and turnover dynamics compared to broadleaved species such as oak, ash, or lime, which differ in longevity, biomass allocation, and mortality regimes. Therefore, observed carbon dynamics and successional trajectories should be interpreted as aggregated patterns across functionally diverse species groups rather than species-specific responses. This aggregation may therefore influence the interpretation of carbon dynamics by smoothing variation in biomass turnover rates and productivity among species, particularly in four-phase forests where successor diversity is highest. Similarly, successional trajectories derived from colonizer–successor contrasts reflect dominant functional tendencies rather than detailed species-level pathways. Distinguishing finer ontogenetic sub-strategies within successor groups represents an important direction for future research and would require higher-resolution inventory or trait-based datasets.

#### 4.1.3. Age Structure of Tree Growing Stock

**Figure 4:**
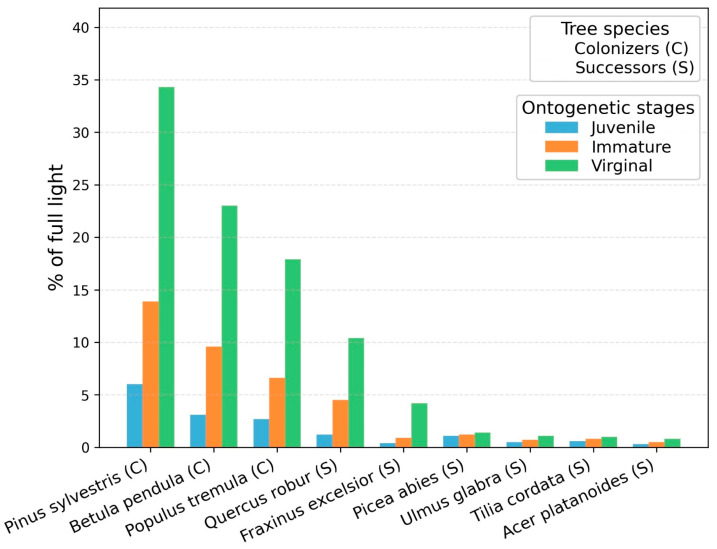
Light thresholds of hemiboreal tree species across ontogenetic stages. Juvenile, immature, and virginal light-requirement values are shown for major hemiboreal tree species. Thresholds are based on published measurements in Evstigneev & Korotkova [64] and Petrokas et al. [8].

Forest development itself encompasses the temporal progression of structure and composition as vegetation stratifies into emergent, canopy, subcanopy, and understory layers, creating diverse ecological niches [37,62]. Stand growth emerges from the combined effects of tree ontogeny, regeneration strategies, growth rates, and mortality, traits that reflect adaptive responses across forest development. As trees progress from juvenile to mature stages, relative growth rates decline, and competition for light, nutrients, and space intensifies, resulting in natural self-thinning [37,63]. Light-threshold differences among species and ontogenetic stages (Figure 4) illustrate how colonizers and successors partition regeneration niches across juvenile, immature, and virginal phases [45].

Ontogenetic age, rather than chronological age, provides a more ecologically meaningful indicator of stand development because it reflects physiological transitions, biomass allocation patterns, and carbon-sequestration capacity [8]. These stage-related shifts are tightly linked to crown architecture, shading tolerance, and life-cycle timing [37,64]. However, direct measurement of ontogenetic stages is not available within the LNFI framework, which is structured around stand age and growing-stock attributes. As a result, chronological age classes are used here as an operational proxy for underlying ontogenetic transitions. While this proxy does not capture the full variability of individual tree development, consistent age-related patterns in growth, mortality, and species composition allow chronological classes to approximate broad ontogenetic phases at the stand level. Accordingly, age-structured analyses presented in this study should be interpreted as representing aggregated expressions of ontogenetic dynamics across stands, rather than exact mappings of individual-level developmental stages.

As tree size generally increases along ontogenetic pathways, volume growth and growing-stock composition serve as indirect indicators of successional position. While traditionally used to evaluate stand productivity [54,63,65], these metrics acquire greater ecological relevance when interpreted through species’ adaptive strategies, demographic patterns, and life-history traits [40,57]. In this context, age-structured growing-stock data provide insight into successional trajectories and the adaptive capacity of forest communities [66]. Mixed-species stands on fertile sites often support both colonizer and successor species with diverse ontogenetic pathways, whereas less favorable sites tend toward single-species, colonizer-dominated structures with reduced ontogenetic and functional diversity [57].

### 4.2. Materials

#### 4.2.1. Study Area and Forest-Typological Context

Hemiboreal forests form a transitional biome between the boreal coniferous and temperate deciduous zones and are characterized by the co-dominance of coniferous and broadleaf tree species across the Baltic States, southern Scandinavia, and parts of northwestern Russia, Canada, and the USA [43]. This study focuses on Lithuania, where previous work has shown that more than half of forest stands are dominated by a single tree species under a 75% dominance threshold, reflecting long-term management and site-conditioned successional pathways [53].

We used observations from the LNFI, a long-term, standardized, GIS-integrated monitoring program covering all forests of Lithuania [63,67]. The LNFI employs a systematic cluster design with four permanent circular plots (500 m^2^) per cluster, spaced 250 m apart, yielding approximately 5600 permanent plots and a sampling intensity of ~1 plot per 400 ha; field measurements are collected annually on a five-year cycle [63]. Measurements include tree growing stock, gross increment, mortality, fellings, and site and soil descriptors, with reported national precisions of ±1.1% for forest area, ±1.5/1.0% for total/mean growing stock volume, and ±1.4/0.9% for total/mean gross volume increment. Tree-level attributes (species, DBH, height, crown height) are scaled to stand and national levels using established LNFI estimators. The LNFI provides nationally consistent, multi-year measurements suitable for broad-scale ecological analysis and international comparability across national forest inventories, supporting the identification of site-conditioned structural development, species composition, and carbon-balance trends in hemiboreal forests [63,68].

For ecological stratification, we applied the Lithuanian forest typological classification system, grouping stands by (i) forest types defined by canopy dominants and (ii) forest type-series that reflect site conditions [52,69]. Site grouping integrated national soil classifications and the World Reference Base (WRB) system, including Arenosols, Luvisols, Gleysols, Fluvisols, Podzols, and Histosols, along with published summaries of topsoil (0–30 cm) organic-carbon stocks [69,70,71,72,73,74,75]. Within the LNFI framework, the age-structured distribution of growing stock was interpreted as the emergent outcome of ontogenetic strategies operating across site conditions. The Lithuanian forest typological classification, based on potential natural vegetation, soil fertility, and moisture regimes [52], provides the ecological template for distinguishing three-phase (colonizer-dominated) and four-phase (mixed colonizer–successor) successional pathways (see next subsection). Each forest type-series was therefore assigned to a three-phase or four-phase successional pathway consistent with national syntheses and LNFI analyses [53]. Because forest ecosystem functions (i.e., productivity, species mixing, and carbon sequestration) are strongly conditioned by this interaction between site variables and species strategies, reorganizing LNFI observations by ontogenetic groups of colonizers and successors, as well as by typology, allows for a more mechanistic interpretation of stand development than dominant-species forest-type reporting alone.

#### 4.2.2. Soil and Vegetation Typology for Site Grouping

To account for ecological gradients, we distinguished two broad successional pathways based on the dominant forest type-series in Lithuania’s hemiboreal zone [57,76]. This division reflects the country’s two principal zonal formations: (i) mesophytic/hygromesophytic coniferous–broadleaved on nutrient-poor substrates and (ii) mesophytic deciduous broadleaved and coniferous–broadleaved forests on more fertile soils. These categories integrate potential natural vegetation, national typological syntheses, and adaptive life-history groupings of tree species [57,76].

On edaphically constrained (nutrient-poor) soils, development follows a three-phase trajectory (stand initiation (SI), stem exclusion (SE), steady state (SS); Table 6). These include the Cladoniosa, Vacciniosa, Vaccinio-myrtillosa, Myrtillo-sphagnosa, Ledo-sphagnosa, Carico-sphagnosa, Caricosa, Carico-iridosa, and Calamagrostidosa series. They are predominantly associated with Arenosols, Regosols, Podzols, Planosols, and shallow Histosols, where coarse texture and low base saturation restrict nutrient availability [75,77]. Ground layers are typically lichen–moss- or dwarf-shrub dominated, and canopies are formed primarily by gap-colonizing species (Table 6). Successional development on these sites is constrained by regeneration traits adapted to low-resource conditions, resulting in simplified vertical structure and limited cohort diversification [43].

On more fertile soils, development follows a four-phase trajectory (SI, SE, understory reinitiation (UR), SS; Table 6). This category includes the Myrtillosa, Oxalidosa, Myrtillo-oxalidosa, Hepatico-oxalidosa, Oxalido-nemorosa, Aegopodiosa, Carico-mixtoherbosa, Filipendulo-mixtoherbosa, and Urticosa series. These series commonly occur on Cambisols, Luvisols, Gleysols, Fluvisols, and nutrient-rich Arenosols [75]. Higher base status and pH support herb-rich understories and mixed canopies composed of both colonizers and successors (Table 6). National Forest Inventory analyses (2002–2022) indicate that fertile-site stands exhibit greater vertical differentiation, multi-cohort structure, and extended successional progression relative to nutrient-poor sites [53].

Across both categories, forest type-series, WRB soil classes, ontogenetic strategy groups (colonizers vs. successors), and successional phases were treated as integrated descriptors of stand development (Table 6). This successional phase-based framework enables the linkage of ecological typology with adaptive life-history strategies to evaluate site-specific successional dynamics, with soil organic carbon stocks included as contextual indicators of site conditions rather than as components of the analyzed carbon balance.

### 4.3. Analytical Approach

#### 4.3.1. Data Organization and Classification

This study applied a structured workflow to organize and analyze age-structured LNFI data across forest type-series, forest types, ontogenetic strategy groups, and forest trajectories. All steps followed published LNFI procedures and established ecological classifications [8,37,53,63].

Forest stands were assigned to three- or four-phase forest trajectories based on the Lithuanian forest type-series classification and species composition criteria defined in national typology and LNFI documentation. Trajectories followed the taxonomy used in Petrokas et al. [53]. Tree species were grouped into colonizers or successors using trait-based classifications (i.e., shade tolerance and growth strategy) as applied in Petrokas et al. [8]. In Appendix A Table A1, Table A2 and Table A3, tree species contributing more than 0.1% to composition are shown (values represent percentage volume by species); species below 0.01% are reported as 0.00. Less frequent species are retained in the full species tables to preserve probabilistic information on rare and non-neutral species occurrences, which may indicate emerging ecological processes and are important for ecological interpretation.

#### 4.3.2. Age-Structured Variables and Metrics

For each group (forest types, forest type-series, forest trajectories, and C/S categories), LNFI data were summarized by age classes: ≤20, 21–40, 41–60, 61–80, 81–100, 101–120, and ≥121 years. Although the analysis is structured using chronological age classes, these classes are interpreted as proxies for underlying ontogenetic progression at the stand level. This approximation reflects the LNFI data structure, which does not directly record ontogenetic stages but captures variables (e.g., growth, mortality, and species composition) that emerge from ontogenetic processes. The use of age classes therefore enables a consistent, large-scale representation of developmental patterns, while acknowledging that ontogenetic variation within and among stands may introduce additional heterogeneity. Structural metrics extracted included tree density (trees/ha), growing stock volume (V) (m^3^/ha), gross mean volume increment (VI) (m^3^/ha/year), increment rate (VI%) (=VI/V × 100), and tree mortality (expressed as % of VI). These variables follow LNFI methodology and prior analyses [29,63,78,79].

#### 4.3.3. Carbon Estimation in Living Biomass

Above-ground biomass (AGB), below-ground biomass (BGB), total carbon stock in living biomass (C), and CO_2_-equivalent calculations were derived using standard biomass expansion and carbon fraction methods [80,81,82,83]. Above- and below-ground biomass were estimated using biomass expansion factor (BEF), basic wood density (D), root-to-shoot ratio (R), and carbon fraction (CF) constants. Carbon increment was derived from mean gross volume increments (VI) using the same parameter set.

Carbon stock and carbon increment were calculated from growing stock volume (V) and gross mean volume increment (VI) using Tier 1 biomass-conversion parameters [84] and species-specific constants provided in national inventory guidance [67]. All biomass conversion parameters were applied as fixed coefficients following Tier 1 IPCC guidance and national forest inventory documentation. The following constants were used consistently across all calculations: basic wood density (D) of 0.41 t d.m. m^−3^ for conifers and 0.47 t d.m. m^−3^ for broadleaved species; biomass expansion factor (BEF) of 1.221 for conifers and 1.178 for broadleaves; root-to-shoot ratio (R) of 0.26 for conifers and 0.19 for broadleaves; and carbon fraction (CF) of 0.51 for conifers and 0.48 for broadleaves. These values follow IPCC [84] Volume 4 Tier 1 default parameters and are consistent with national inventory practice [63,78,79,84]. The following equations were applied:

AGB = V × D × BEF × A

BGB = AGB × R

C = AGB × (1 + R) × CF

CO_2_ = C × 3.67

where:

V—growing stock volume (m^3^/ha)
A—area (ha) in each age class
3.67—carbon-to-CO_2_ conversion factor [84]

Accordingly, all carbon stock and CO_2_ accumulation values reported in Table 1, Table 2 and Table 3 include both above- and below-ground biomass. Below-ground carbon is embedded in every reported figure through the (1 + R) term in the carbon equation, where R is the root-to-shoot ratio applied by species group.

It is important to distinguish between gross mean volume increment (VI) and CO_2_ accumulation in living biomass. While VI represents gross biomass production, CO_2_ accumulation is derived from the net change in growing stock volume and therefore incorporates the combined effects of growth, mortality, and harvesting removals. Consequently, increasing growing stock volume with stand age can occur simultaneously with declining or negative CO_2_ accumulation, particularly in later developmental stages where mortality and removals exceed net growth. These variables therefore describe different aspects of forest dynamics and are not expected to display identical trends.

CO_2_ accumulation in living biomass was calculated by converting annual change in mean growing stock volume to biomass and carbon using the same D, BEF, R, and CF parameters. Within the LNFI framework, annual change in growing stock volume represents net change, incorporating gross increment, natural mortality, and removals due to harvesting. Accordingly, estimated CO_2_ accumulation reflects net changes in living biomass carbon under existing management conditions. Negative values therefore indicate a net reduction in living biomass carbon resulting from the combined effects of mortality and harvesting, rather than ecological processes alone. All conversions were based on LNFI 2024 growing stock volume change data, and biomass-conversion and carbon-fraction parameters followed national inventory documentation and IPCC Tier 1 guidance [67,84]. Net CO_2_ loss refers to a negative annual change in carbon stock in living biomass derived from growing stock volume dynamics. It should be noted that carbon accounting in this study is restricted to living biomass pools (above- and below-ground). Other major forest carbon pools are not included due to data limitations within the LNFI framework. Soil organic carbon data are presented to characterize site conditions and ecological context but are not integrated into the carbon-balance calculations. Consequently, reported CO_2_ accumulation and loss values represent changes in living biomass only and should not be interpreted as a full ecosystem carbon balance. Carbon increment estimates are therefore directly derived from volume increment using these fixed conversion parameters and should be interpreted as generalized approximations of biomass carbon change consistent with Tier 1 accounting approaches.

#### 4.3.4. Analytical Frameworks and Comparison

Two grouping systems were applied to the same LNFI dataset: (i) dominant-species (forest-type) reporting and (ii) typology-based (forest type-series) classification with three- or four-phase trajectories. The three complementary data classification frameworks applied to age-structured observations from the LNFI were compared by contrasting how identical LNFI stand-level indicators (listed below) were distributed when grouped (i) by forest-type and (ii) by forest type-series and forest trajectory. Only the data grouping system differed; all underlying LNFI variables, age-class boundaries, and calculation procedures remained consistent across all frameworks.

Volume growth-oriented management reporting framework (Results Section 2.1)

In this system, stands were grouped by their dominant tree species, i.e., forest types. For each dominant species group, the following stand-level indicators were calculated along the age gradient:

tree density (trees/ha),
mean growing stock volume (V),
annual gross mean volume increment (VI),
increment rate (VI% from V),
tree mortality (expressed as % of VI),
annual CO_2_ accumulation in living biomass (calculated from change in V).

These indicators were summarized separately for stands dominated by colonizer (C) and successor (S) tree species.

Biodiversity- and structure-oriented typology-based framework (Results Section 2.2)

In this system, the same LNFI data were reorganized according to Lithuanian forest type-series, representing ecological site conditions defined by soil fertility and moisture regime. Each series was assigned to either a three-phase or four-phase forest trajectory. Within these trajectories, stand structure and carbon-related variables were calculated using the same indicators listed above, and summarized across the same stand-age classes. This classification also enabled the extraction of colonizer and successor strategy components within each forest type-series.

Climate-oriented (carbon dynamics) framework (Results Section 2.3)

In addition, carbon-related indicators, including CO_2_ accumulation in living biomass derived from changes in growing stock volume, were analyzed to evaluate climate-relevant dynamics across forest trajectories and stand-age classes.

#### 4.3.5. Trajectory-Level Interpretation and Assumptions

Within each forest trajectory and forest type-series, structural and carbon variables were summarized by stand-age class and by colonizer/successor species components. Because the study is based on aggregated LNFI cross-sectional summaries rather than plot-level datasets, formal inferential statistics were not applied. Instead, age-class comparisons follow a space-for-time substitution approach, in which stands of different chronological ages are interpreted as representing generalized stages along successional trajectories. However, because the analysis is based on cross-sectional inventory data rather than repeated measurements of the same stands, these comparisons do not represent direct temporal development. Observed differences among stand-age classes may therefore reflect a combination of successional processes and spatial variation in site conditions, species composition, historical management practices, disturbance regimes, and climatic influences. Consequently, the identified patterns should be interpreted as large-scale, aggregated tendencies in forest development across typological and ontogenetic groupings, rather than as deterministic or linear representations of stand-level temporal dynamics.

## 5. Conclusions

This study provides a process-based framework for interpreting large-scale patterns of forest development using Lithuanian National Forest Inventory data reorganized by forest type-series and ontogenetic strategies. By integrating succession theory with typology-based classification, the approach enables the identification of contrasting forest developmental trajectories that are not detectable using dominant-species reporting alone.

The results indicate that forest development in the hemiboreal ecoregion is structured by interacting ontogenetic processes and site conditions, giving rise to distinct successional and biomass-turnover patterns across forest trajectories. Fertile forest sites were characterized by higher early–mid-age productivity and more dynamic structural reorganization, whereas nutrient-poor forest sites exhibited slower but more persistent developmental pathways. Correspondingly, living biomass carbon stocks differed in the timing and intensity of biomass turnover, with earlier transitions to negative net CO_2_ stocks in fertile sites and more prolonged carbon accumulation in nutrient-poor forest sites.

Importantly, living biomass carbon stocks derived from growing stock reflect net fluxes rather than cumulative carbon stocks. Consequently, increasing growing stock volume may occur simultaneously with negative CO_2_ accumulation where mortality and harvesting exceed growth. This distinction highlights the limitation of using growing stock volume alone as an indicator of carbon dynamics or ecosystem conditions.

The observed divergence between living biomass carbon stocks and compositional and structural complexity, i.e., high growing stock persisting in structurally simplified, colonizer-dominated three-phase stands, versus lower standing volume but greater species and cohort diversity in four-phase stands, indicates that carbon stocks alone should not be treated as a proxy for biodiversity. Forest structure, species composition, and successional dynamics should therefore be considered jointly, alongside carbon metrics, when assessing forest condition and management outcomes.

Given the cross-sectional nature of the LNFI dataset and the use of a space-for-time substitution approach, the identified patterns represent generalized, large-scale tendencies rather than direct temporal trajectories of individual stand development. In addition, carbon estimates are restricted to living biomass pools and are based on generalized biomass-conversion parameters, consistent with the IPCC’s Tier 1 accounting approaches.

Overall, the proposed framework provides a practical tool for integrating structural, carbon, and ecological indicators within national forest inventory systems. By linking typology, ontogenetic strategies, and carbon dynamics, it supports more ecologically informed forest management and contributes to improved assessment of forest condition in the context of climate mitigation and biodiversity-oriented policy objectives.

## Figures and Tables

**Table 1:** Volume growth-oriented forest inventory indicators across age classes for colonizer and successor forest types.

Stand-Level Data	Stand Age Classes							LNFI 2024 Mean
	≤20	21–40	41–60	61–80	81–100	101–120	≥121	
**Colonizers**: Scots pine, silver and downy birch, Eurasian aspen, gray alder, black alder								
Area (1000 ha)	287.0	299.4	325.3	408.0	194.4	96.9	39.3	1650.2
Tree density (trees/ha)	3017	1941	1264	951	955	837	824	1543
Mean growing stock volume (V) (m^3^/ha)	56.6	187.1	299.2	360.1	408.7	439.1	446.7	276.4
Gross mean volume increment (VI) (m^3^/ha/year)	6.1	11.5	10.1	9.2	8.5	7.9	7.1	9.0
Volume increment rate (VI% from V)	10.7	6.2	3.4	2.5	2.1	1.8	1.6	3.3
Tree mortality (% from VI)	7.3	12.6	29.3	29.2	33.1	33.9	38.4	24.6
CO_2_ accumulation in living biomass (t/ha/year)	6.1	9.6	2.9	0.0	−2.2	−3.0	−2.3	2.6
**Successors**: English oak, European ash, European hornbeam, Norway spruce, European field elm, wych elm, European white elm, small-leaved lime, Norway maple								
Area (1000 ha)	82.3	118.2	101.1	90.8	61.8	25.4	14.3	493.8
Tree density (trees/ha)	2521	1968	1144	910	836	728	662	1454
Mean growing stock volume (V) (m^3^/ha)	42.6	177.8	292.4	356.7	398.7	407.4	420.5	258.1
Gross mean volume increment (VI) (m^3^/ha/year)	3.7	12.3	10.3	9.5	8.7	8.5	9.2	9.2
Volume increment rate (VI% from V)	8.6	6.9	3.5	2.7	2.2	2.1	2.2	3.6
Tree mortality (% from VI)	2.6	6.7	27.9	36.7	41.5	33.8	48.2	25.0
CO_2_ accumulation in living biomass (t/ha/year)	4.4	11.3	2.3	−5.0	−3.1	−1.8	−7.3	2.0

**Table 2:** Biodiversity-oriented forest inventory indicators across age classes for colonizer and successor forest types in three-phase forests: cl, v, vm, msp/s, cal/s, lsp/s, csp/s, c/s, cir/s.

Stand-Level Data	Stand Age Classes							LNFI 2024 Mean
	≤20	21–40	41–60	61–80	81–100	101–120	≥121	
**Colonizers (pioneers)**								
Area (1000 ha)	59.4	79.8	122.6	179.2	104.5	59.7	24.1	629.3
Tree density (trees/ha)	2476	1866	1228	911	874	817	728	1220
Mean growing stock volume (V) (m^3^/ha)	42.6	146.6	246.6	319.3	378.9	399.3	403.8	277.8
Gross mean volume increment (VI) (m^3^/ha/year)	4.0	9.4	8.3	8.1	7.7	7.1	6.3	7.7
Volume increment rate (VI% from V)	9.3	6.4	3.4	2.5	2.0	1.8	1.6	2.8
Tree mortality (% from VI)	5.5	12.1	28.6	27.9	28.5	29.9	27.4	25.3
CO_2_ accumulation in living biomass (t/ha/year)	1.8	8.5	4.7	2.9	0.8	−3.4	−2.1	2.6
**Successors (post-pioneers)**								
Area (1000 ha)	3.6	13.3	14.5	11.2	5.3	3.2	1.2	52.4
Tree density (trees/ha)	1675	1733	1250	1148	968	622	880	1306
Mean growing stock volume (V) (m^3^/ha)	18.8	129.5	214.5	287.7	372.7	357.5	320.6	222
Gross mean volume increment (VI) (m^3^/ha/year)	0.8	9.4	8.2	7.4	6.9	6.3	7.6	7.6
Volume increment rate (VI% from V)	4.5	7.3	3.8	2.6	1.9	1.8	2.4	3.4
Tree mortality (% from VI)	0.7	7.2	25.2	27.6	46.9	39.2	20.2	22.8
CO_2_ accumulation in living biomass (t/ha/year)	−4.3	6.6	5.7	2.6	2.7	−7.6	−12.4	2.9
**Three-phase pathway (all tree species)**								
Area (1000 ha)	63.0	93.1	137.2	190.4	109.8	62.9	25.3	681.7
Tree density (trees/ha)	2430	1847	1230	925	879	808	735	1226
Mean growing stock volume (V) (m^3^/ha)	41.3	144.2	243.2	317.5	378.6	397.2	399.9	273.6
Gross mean volume increment (VI) (m^3^/ha/year)	3.8	9.4	8.3	8.1	7.7	7.0	6.4	7.7
Volume increment rate (VI% from V)	9.2	6.5	3.4	2.5	2.0	1.8	1.6	2.8
Tree mortality (% from VI)	5.4	11.3	28.3	27.9	29.3	30.4	27.0	25.2
CO_2_ accumulation in living biomass (t/ha/year)	1.5	8.2	4.8	2.9	0.9	−3.6	−2.7	2.6

**Table 3:** Biodiversity-oriented forest inventory indicators across age classes for colonizer and successor forest types in four-phase forests: m, ox, mox, fil/s, ur/s, hox, oxn, aeg, cmh.

Stand-Level Data	Stand Age Classes							LNFI 2024 Mean
	≤20	21–40	41–60	61–80	81–100	101–120	≥121	
**Colonizers (pioneers)**								
Area (1000 ha)	227.6	219.6	202.7	228.8	89.9	37.2	15.3	1020.9
Tree density (trees/ha)	3159	1969	1286	983	1048	868	977	1742
Mean growing stock volume (V) (m^3^/ha)	60.3	201.9	331.0	392.1	443.3	503.2	514.4	275.5
Gross mean volume increment (VI) (m^3^/ha/year)	6.6	12.3	11.2	10.0	9.3	9.3	8.2	9.9
Volume increment rate (VI% from V)	11.0	6.1	3.4	2.6	2.1	1.9	1.6	3.6
Tree mortality (% from VI)	7.6	12.8	29.5	29.8	37.2	38.7	52.0	24.2
CO_2_ accumulation in living biomass (t/ha/year)	7.2	10.0	1.8	−2.1	−5.5	−2.2	−2.6	2.6
**Successors (post-pioneers)**								
Area (1000 ha)	78.6	104.8	86.5	79.6	56.5	22.3	13.1	441.4
Tree density (trees/ha)	2560	1998	1126	876	824	743	642	1471
Mean growing stock volume (V) (m^3^/ha)	43.7	183.9	305.4	366.4	401.1	414.4	429.7	262.4
Gross mean volume increment (VI) (m^3^/ha/year)	3.8	12.7	10.6	9.8	8.9	8.8	9.4	9.4
Volume increment rate (VI% from V)	8.7	6.9	3.5	2.7	2.2	2.1	2.2	3.6
Tree mortality (% from VI)	2.6	6.6	28.2	37.5	41.1	33.3	50.6	25.2
CO_2_ accumulation in living biomass (t/ha/year)	4.9	11.9	1.7	−6.0	−3.6	−0.9	−6.7	1.9
**Four-phase pathway (all tree species)**								
Area (1000 ha)	306.2	324.4	289.2	308.3	146.3	59.4	28.4	1462.4
Tree density (trees/ha)	3005	1978	1238	955	961	821	822	1660
Mean growing stock volume (V) (m^3^/ha)	56.0	196.1	323.3	385.5	427.0	469.9	475.3	271.5
Gross mean volume increment (VI) (m^3^/ha/year)	5.9	12.4	11.0	9.9	9.1	9.1	8.8	9.7
Volume increment rate (VI% from V)	10.5	6.3	3.4	2.6	2.1	1.9	1.8	3.6
Tree mortality (% from VI)	6.9	10.7	29.1	31.8	38.6	36.8	51.3	24.5
CO_2_ accumulation in living biomass (t/ha/year)	6.6	10.6	1.8	−3.2	−4.8	−1.7	−4.5	2.3

**Table 4:** Species composition of growing stock by forest-type and forest type-series categories. Percent volumes of tree species are shown. Letter codes indicate tree species: a, *Quercus robur*; b, *Betula pendula* and *Betula pubescens*; bt, *Alnus incana*; d, *Populus tremula*; e, *Picea abies*; g, *Ulmus glabra*; j, *Alnus glutinosa*; k, *Acer platanoides*; l, *Tilia cordata*; p, *Pinus sylvestris*; s, *Ulmus minor*; sb, *Carpinus betulus*; u, *Fraxinus excelsior*; vn, *Ulmus laevis*.

Categories	Prevailing Tree Species in Stand									Total Mean
	p	e	B	d	j	bt	a	u	Other	
Colonizers (p, b, d, bt, j)	82 p 10 e 5 b	- - -	62 b 15 e 5 j	63 d 12 e 11 b	73 j 11 b 7 e	71 bt 8 b 5 e	- - -	- - -	- - -	44 p 18 b 12 j
Area (1000 ha)	673.0	-	435.5	162.0	248.0	131.7	-	-	-	1650.2
Successors (a, u, sb, e, s, g, vn, l, k)	- - -	74 e 8 b 6 p					58 a 13 e 8 b	46 u 12 e 7 a	33 l 25 k 8 a	63 e 9 a 8 b
Area (1000 ha)	0.0	399.0	0.0	0.0	0.0	0.0	54.7	9.9	30.2	493.8
Colonizers (p, b, d, bt, j) in three-phase forests: cl, v, vm, msp (msps), cal (cals), lsp (lsps), csp (csps), c (cs), cir (cirs)	88 p 7 e 4 b	- - -	69 b 10 e 9 j	70 d 16 b 8 e	79 j 11 b 7 e	76 bt 15 j 8 b	- - -	- - -	- - -	69 p 11 j 10 b
Area (1000 ha)	435.5	0.0	95.7	7.0	87.3	3.7	0.0	0.0	0.0	629.3
Colonizers (p, b, d, bt, j) in four-phase forests: m, ox, mox, fil (fils), ur (urs), hox, oxn, aeg, cmh	75 p 14 e 6 b	- - -	61 b 16 e 6 d	62 d 12 e 10 b	69 j 11 b 8 e	71 bt 8 b 5 e	- - -	- - -	- - -	28 p 23 b 13 e
Area (1000 ha)	237.6	0.0	339.8	155.0	160.7	128.0	0.0	0.0	0.0	1020.9
Successors (a, u, sb, e, s, g, vn, l, k) in three-phase forests: m, ox, mox, fil (fils), ur (urs), hox, oxn, aeg, cmh	- - -	71 e 13 p 9 b	- - -	- - -	- - -	- - -	43 a 28 b 27 p	- - -	- - -	71 e 13 p 10 b
Area (1000 ha)	0.0	51.3	0.0	0.0	0.0	0.0	1.1	0.0	0.0	52.4
Successors (a, u, sb, e, s, g, vn, l, k) in four-phase forests: cl, v, vm, msp (msps), cal (cals), lsp (lsps), csp (csps), c (cs), cir (cirs)	- - -	74 e 8 b 5 p	- - -	- - -	- - -	- - -	58 a 13 e 8 b	46 u 12 e 7 a	33 l 25 k 8 a	62 e 10 a 8 b
Area (1000 ha)	0.0	347.7	0.0	0.0	0.0	0.0	53.6	9.9	30.2	441.4
Three-phase pathway (all tree species): cl, v, vm, msp (msps), cal (cals), lsp (lsps), csp (csps), c (cs), cir (cirs)	88 p 7 e 4 b	71 e 13 p 9 b	69 b 10 e 9 j	70 d 16 b 8 e	79 j 11 b 7 e	76 bt 15 j 8 b	43 a 28 b 27 p	- - -	- - -	66 p 11 e 11 j
Area (1000 ha)	435.5	51.3	95.7	7.0	87.3	3.7	1.1	0.0	0.0	681.7
Four-phase pathway (all tree species): m, ox, mox, fil (fils), ur (urs), hox, oxn, aeg, cmh	75 p 14 e 6 b	74 e 8 b 5 p	61 b 16 e 6 d	62 d 12 e 10 b	69 j 11 b 8 e	71 bt 8 b 5 e	58 a 13 e 8 b	46 u 12 e 7 a	33 l 25 k 8 a	27 e 21 p 18 b
Area (1000 ha)	237.6	347.7	339.8	155.0	160.7	128.0	53.6	9.9	30.2	1462.4

**Table 6:** Forest typological classification and indicative estimates of area and soil organic carbon (SOC) stocks. SOC values are presented as contextual site indicators and are not included in the living biomass carbon balance analysis.

Forest Type-Series *	Soil Types **	Ontogenetic Strategies of Tree Species ***	Stand Successional Phases ****	Area, 1000 ha	SOC Stock (0–30 cm), t C/ha *****	Total SOC Stock (0–30 cm), Mt C
Cladoniosa (cl)	AR, RG	C (p)	SI SE SS	0.9	55.7	0.1
Vacciniosa(v)	AR, PZ, RG	C (p bk)	SI SE SS	63.2	81.1	5.1
Vaccinio-myrtillosa (vm)	AR, PZ	C (p bk d)	SI SE SS	335.4	81.1	27.2
Myrtillo-sphagnosa (msp/msps)	GL, PZ	C (p bp)	SI SE SS	15.4	94.9	1.5
Ledo-sphagnosa (lsp/lsps)	HSf	C (p)	SI SE SS	38.6	150.5	5.8
Carico-sphagnosa (csp/csps)	HSf-s	C (p bp)	SI SE SS	85.8	150.5	12.9
Caricosa (c/cs)	HSs	C (bp j)	SI SE SS	168.0	150.5	25.3
Carico-iridosa (cir/cirs)	HSs	C (bp j)	SI SE SS	35.0	180.5	6.3
Calamagrostidosa (cal cals)	GL	C (bp bk j)	SI SE SS	45.7	96.1	4.4
Myrtillosa (m)	AR, PL, PZ	C (p bk d) S (e)	SI SE UR SS	63.6	81.1	5.2
Oxalido-nemorosa (oxn)	LV, CM, FL	C (bp bk d bt j) S (a u sb e g vn l k)	SI SE UR SS	345.1	109.9	37.9
Carico-mixtoherbosa (cmh)	LV, CM	C (bp bk d bt j) S (a u e g vn l k)	SI SE UR SS	45.1	109.9	5.0
Aegopodiosa (aeg)	LV, CM	C (bp bk d bt j) S (a u e g vn l k)	SI SE UR SS	7.5	108.9	0.8
Myrtillo-oxalidosa (mox)	AR, LV, RT, PL, CM	C (p bk d) S (a sb e)	SI SE UR SS	311.2	91.4	28.4
Oxalidosa (ox)	AR, LV, RT, PL, CM, FL	C (p bk d) S (a sb e)	SI SE UR SS	385.9	91.4	35.3
Hepatico-oxalidosa (hox)	AR, LV, RT, PL, CM, FL	C (p bk d bt) S (a u sb e g vn l k)	SI SE UR SS	126.8	90.3	11.4
Filipendulo-mixtoherbosa (fil/fils)	GL, FL	C (bp bk j) S (u)	SI SE UR SS	84.8	102.3	8.7
Urticosa (ur/urs)	GL	C (bp bk gt gb bt j) S (u)	SI SE UR SS	3.0	91.8	0.3

* Ground layer flora (codes); *s*—index of drained (*siccatum*) forest types. ** RT—Retisols, AR—Arenosols, CM—Cambisols, FL—Fluvisols, GL—Gleysols, HSf—Fibric Histosols, HSf-s—Terri-Fibric Histosols, HSs—Pachiterric Histosols, LV—Luvisols, PL—Planosols, PZ—Podzols, RG—Regosols. *** Tree species: colonizers (C)—*Pinus sylvestris* (p), *Betula pubescens* (bp), *Betula pendula* (bk), *Salix fragilis* (gt), *Salix alba* (gb), *Populus tremula* (d), *Alnus incana* (bt), *Alnus glutinosa* (j); successors (S)—*Quercus robur* (a), *Fraxinus excelsior* (u), *Carpinus betulus* (sb), *Picea abies* (e), *Ulmus glabra* (g), *Ulmus laevis* (vn), *Tilia cordata* (l), *Acer platanoides* (k). **** SI—stand initiation, SE—stem exclusion, UR—understorey reinitiation, SS—steady state. ***** Average SOC stock estimates at the plot level are approximate and derived from the midpoints of reported SOC range values.

## Data Availability

We obtained the data from the Lithuanian State Forest Service. Requests to access the datasets should be directed to Gintaras Kulbokas, Lithuanian State Forest Service.

## Appendix A

**Table A1:** Age-class structure of tree growing stock by colonizer and successor categories of forest types.

Stand-Level Data	Stand Age Classes							LNFI 2024 Mean
	≤20	21–40	41–60	61–80	81–100	101–120	≥121	
(a) Colonizers: Scots pine (p), silver and downy birch (b), Eurasian aspen (d), gray alder (bt), black alder (j)								
Species composition by growing stock volume *	24.40 b	28.90 b	28.40 p	49.30 p	60.30 p	70.40 p	78.40 p	43.90 p
	20.00 j	16.70 j	22.10 b	17.90 b	14.50 e	15.70 e	15.80 e	17.90 b
	17.50 d	15.10 d	19.80 j	11.00 e	12.20 b	7.70 b	2.80 b	11.50 j
	12.00 p	14.10 bt	9.50 d	10.80 j	5.10 d	2.50 j	1.00 a	10.80 e
	9.80 bt	11.70 p	9.00 bt	6.10 d	4.70 j	1.30 a	0.90 j	7.50 d
	6.50 e	7.30 e	7.00 e	1.60 bt	1.20 a	1.10 d	0.60 k	4.60 bt
	4.90 a	1.60 bl	1.20 a	1.40 a	0.60 l	0.50 k	0.20 d	1.40 a
	1.30 bl	1.60 a	0.90 k	0.60 k	0.40 bt	0.50 l	0.10 l	0.60 k
	1.00 l	0.80 k	0.50 l	0.60 l	0.30 k	0.10 u	0.10 sb	0.50 l
	0.90 k	0.80 u	0.40 bl	0.30 u	0.30 u	0.10 sb	0.10 bl	0.40 bl
	0.80 u	0.40 gl	0.40 u	0.10 bl	0.20 g	0.10 bt	0.02 gl	0.40 u
	0.40 vn	0.40 l	0.30 gl	0.10 sb	0.10 bl	0.10 g		0.10 gl
	0.30 gl	0.20 g	0.20 g	0.10 g	0.03 sb	0.05 bl		0.10 g
	0.10 sb	0.10 vn	0.10 vn	0.04 s	0.03 s	0.03 s		0.10 sb
	0.03 g	0.08 t	0.10 t	0.04 vn	0.01 gl			0.10 vn
	0.02 t	0.07 sb	0.10 sb	0.02 gl	0.01 ku			0.03 s
	0.01 s	0.04 ar	0.04 m	0.00 ku				0.03 t
	0.00 ku	0.03 s	0.03 s	0.00 ar				0.01 ar
	0.00 ar	0.02 pb	0.02 ar	0.00 pb				0.01 m
		0.00 ku	0.01 ku					0.01 ku
			0.00 kp					0.00 pb
			0.00 kk					0.00 kp
			0.00 pb					0.00 kk
			0.00 ka					0.00 ka
(b) Successors: English oak (a), European ash (u), European hornbeam (sb), Norway spruce (e), European field elm (s), wych elm (g), European white elm (vn), small-leaved lime (l), Norway maple (k)								
Species composition by growing stock volume *	38.70 e	72.90 e	74.60 e	60.00 e	60.70 e	48.20 e	33.50 a	62.70 e
	19.60 a	8.30 b	7.80 b	8.80 a	8.30 a	16.00 a	30.70 e	9.30 a
	10.90 b	5.60 a	4.80 a	8.60 b	8.00 b	8.80 p	7.80 k	8.10 b
	8.50 p	2.50 p	3.00 p	5.90 p	7.50 p	7.80 b	6.40 l	5.20 p
	6.30 d	2.30 d	2.20 bt	4.40 l	4.00 d	5.20 l	5.70 b	3.10 d
	4.00 k	2.30 j	1.80 j	3.10 k	2.90 l	4.90 d	4.50 d	2.90 l
	3.10 l	1.70 bt	1.50 d	3.00 d	2.20 j	2.40 sb	3.60 p	2.30 k
	2.90 u	1.20 k	1.20 k	1.70 j	2.10 k	2.00 j	2.80 u	2.00 j
	2.20 j	0.80 l	1.10 l	1.70 bt	1.40 sb	1.90 k	1.60 j	1.50 bt
	2.00 bl	0.70 u	0.80 u	1.20 u	1.10 u	1.50 u	1.60 sb	1.20 u
	0.70 bt	0.60 bl	0.40 bl	0.40 sb	0.90 bt	0.50 vn	1.00 g	0.70 sb
	0.60 gl	0.40 gl	0.30 g	0.40 g	0.40 g	0.50 g	0.60 bt	0.40 g
	0.30 g	0.30 sb	0.10 gl	0.20 vn	0.30 vn	0.30 bt	0.10 bl	0.30 bl
	0.10 sb	0.20 g	0.10 sb	0.20 bl	0.10 bl	0.10 bl		0.20 vn
	0.00 ed	0.10 vn	0.10 vn	0.10 gl	0.02 s	0.02 s		0.20 gl
		0.00 s	0.03 s	0.03 s				0.02 s
		0.00 ku	0.02 vk					0.00 vk
		0.00 t	0.02 ka					0.00 ka
			0.00 ku					0.00 ku
								0.00 t
								0.00 ed

* Species > 0.1% are shown; <0.01% reported as 0.00 (values represent percentage volume by species). Species lists include all taxa observed across LNFI datasets; the presence and relative contribution of individual species may vary among Appendix tables due to differences in site conditions, forest types, and age-class distributions. Letter codes denote tree species: a—*Quercus robur*, b—*Betula pendula* and *Betula pubescens*, bl—*Salix caprea*, bt—*Alnus incana*, d—*Populus tremula*, e—*Picea abies*, ed—*Picea pungens*, g—*Ulmus glabra*, gl—*Salix fragilis* and *Salix alba*, j—*Alnus glutinosa*, k—*Acer platanoides*, ka—*Aesculus hippocastanum*, kk—other hardwoods, kp—*Pinus mugo*, ku—*Acer negundo*, l—*Tilia cordata*, m—*Larix* sp., p—*Pinus sylvestris*, pb—*Pinus banksiana*, s—*Ulmus minor*, sb—*Carpinus betulus*, t—*Populus* sp., u—*Fraxinus excelsior*, vk—*Robinia pseudoacacia*, and vn—*Ulmus laevis*.

**Table A2:** Age-class structure of tree growing stock by colonizer and successor categories of forest type-series.

Stand-Level Data	Stand Age Classes							LNFI 2024 Mean
	≤20	21–40	41–60	61–80	81–100	101–120	≥121	
(c) Colonizers (p, b, d, bt, j) in three-phase forests: cl, v, vm, msp/s, cal/s, lsp/s, csp/s, c/s, cir/s								
Species composition by growing stock volume *	42.20 p	30.80 b	47.50 p	74.10 p	80.00 p	82.00 p	84.80 p	69.10 p
	31.70 j	29.80 p	27.20 j	9.10 j	9.30 e	11.50 e	11.40 e	11.10 j
	13.80 b	25.20 j	18.00 b	8.90 b	5.90 b	3.40 b	2.70 b	10.20 b
	4.80 e	6.00 d	3.90 e	6.40 e	3.80 j	2.60 j	0.60 a	7.40 e
	3.40 bt	4.50 e	1.60 d	0.80 d	0.50 d	0.30 a	0.50 j	1.10 d
	1.90 d	2.00 bt	1.00 bt	0.40 a	0.30 a	0.10 d	0.01 k	0.40 bt
	1.00 a	0.50 bl	0.20 e	0.20 bt	0.10 bt	0.10 k		0.30 a
	0.60 bl	0.40 gl	0.20 gl	0.10 k	0.10 bl	0.03 l		0.01 bl
	0.30 k	0.40 a	0.10 l	0.04 l	0.05 l	0.01 bl		0.01 k
	0.10 u	0.20 u	0.10 k	0.02 bl	0.03 k	0.00 bt		0.01 gl
	0.10 t	0.10 g	0.10 ar	0.02 gl	0.02 gl			0.05 l
	0.00 ar	0.10 pb	0.03 u	0.01 sb				0.02 u
		0.10 k	0.02 bl	0.01 u				0.01 ar
		0.04 l	0.02 kp	0.01 ku				0.01 g
			0.02 g	0.00 pb				0.01 pb
			0.00 pb	0.00 g				0.00 sb
			0.00 t					0.00 kp
								0.00 t
								0.00 ku
(d) Colonizers (p, b, d, bt, j) in four-phase forests: m, ox, mox, fil/s, ur/s, hox, oxn, aeg, cmh								
Species composition by growing stock volume *	26.40 b	28.50 b	23.90 b	33.40 p	40.80 p	55.60 p	70.60 p	28.20 p
	20.40 d	17.50 d	19.80 p	23.60 b	19.70 e	21.00 e	21.30 e	22.60 b
	17.80 j	17.30 bt	16.40 j	13.90 e	18.60 b	13.10 b	2.90 b	12.80 e
	11.00 bt	14.50 j	13.10 d	11.90 j	9.70 d	2.60 a	1.50 a	11.80 j
	6.80 e	8.00 e	12.70 bt	9.60 d	5.70 j	2.30 j	1.40 j	11.50 d
	6.40 p	6.90 p	8.40 e	2.60 bt	2.10 a	2.30 d	1.30 k	7.20 bt
	5.60 a	1.90 bl	1.60 a	2.10 a	1.10 l	1.20 l	0.40 d	2.10 a
	1.40 bl	1.90 a	1.20 k	0.90 k	0.70 bt	1.10 k	0.30 l	1.00 k
	1.20 l	1.00 k	0.70 l	0.90 l	0.60 k	0.20 u	0.20 sb	0.90 l
	1.00 k	0.90 u	0.60 bl	0.50 u	0.50 u	0.20 sb	0.10 bl	0.60 bl
	1.00 u	0.50 l	0.50 u	0.20 bl	0.30 g	0.20 bt	0.04 gl	0.60 u
	0.50 vn	0.50 gl	0.30 gl	0.20 sb	0.10 bl	0.10 g		0.20 g
	0.40 gl	0.20 g	0.20 g	0.10 g	0.10 sb	0.10 bl		0.20 gl
	0.10 sb	0.20 vn	0.10 vn	0.10 s	0.10 s	0.10 s		0.10 sb
	0.04 g	0.10 t	0.10 t	0.10 vn	0.02 ku			0.10 vn
	0.02 s	0.10 sb	0.10 sb	0.03 gl	0.01 gl			0.10 s
	0.00 ku	0.04 ar	0.10 m	0.00 ar				0.04 t
		0.04 s	0.04 s	0.00 ku				0.01 m
		0.00 ku	0.02 ku					0.01 ku
			0.00 kk					0.01 ar
			0.00 ka					0.00 kk
								0.00 ka
(e) Successors (a, u, sb, e, s, g, vn, l, k) in four-phase forests: m, ox, mox, fil/s, ur/s, hox, oxn, aeg, cmh								
Species composition by growing stock volume *	38.90 e	73.00 e	74.60 e	59.00 e	59.80 e	46.00 e	35.80 a	61.90 e
	19.90 a	8.00 b	7.20 b	9.70 a	9.00 a	18.00 a	29.20 e	10.20 a
	11.00 b	6.00 a	5.20 a	8.60 b	8.40 b	7.60 p	8.40 k	8.00 b
	7.40 p	2.50 d	2.60 p	5.00 p	6.80 p	7.50 b	6.90 l	4.40 p
	6.40 d	2.20 j	2.30 bt	4.90 l	4.20 d	5.80 l	5.30 b	4.30 d
	4.10 k	1.90 bt	1.80 j	3.50 k	3.20 l	5.30 d	4.70 d	3.20 l
	3.20 l	1.60 p	1.70 d	3.20 d	2.80 k	2.70 sb	2.90 u	2.50 k
	3.00 u	1.30 k	1.30 k	1.90 bt	1.70 j	2.10 k	2.30 p	1.70 j
	2.20 j	0.90 l	1.20 l	1.40 j	1.50 sb	1.80 j	1.70 sb	1.60 bt
	2.10 bl	0.80 u	0.90 u	1.30 u	1.20 u	1.70 u	1.10 j	1.30 u
	0.70 bt	0.70 bl	0.40 bl	0.50 sb	1.00 bt	0.50 vn	1.00 g	0.80 sb
	0.60 gl	0.50 gl	0.40 g	0.40 g	0.40 g	0.50 g	0.60 bt	0.40 g
	0.30 g	0.30 sb	0.10 gl	0.20 vn	0.30 vn	0.40 bt	0.10 bl	0.30 bl
	0.10 sb	0.20 g	0.10 sb	0.20 bl	0.10 bl	0.10 bl		0.20 vn
	0.00 ed	0.10 vn	0.10 vn	0.10 gl	0.02 s	0.02 s		0.20 gl
		0.01 s	0.04 s	0.03 s				0.02 s
		0.00 ku	0.02 vk					0.01 vk
		0.00 t	0.02 ka					0.01 ka
			0.00 ku					0.00 ku
								0.00 t
								0.00 ed
(f) Successors (a, u, sb, e, s, g, vn, l, k) in typical three-phase forests: cl, v, vm, msp/s, cal/s, lsp/s, csp/s, c/s, cir/s								
Species composition by growing stock volume *	63.70 p	72.00 e	74.90 e	69.70 e	71.00 e	66.80 e	52.80 e	70.60 e
	31.70 e	11.70 p	12.80 b	14.00 p	15.70 p	18.00 p	22.80 p	13.90 p
	3.60 b	11.40 b	6.40 p	9.40 b	7.70 j	10.30 b	11.60 b	9.60 b
	0.90 a	2.70 j	2.30 j	5.00 j	4.10 b	3.30 j	9.80 j	4.40 j
		1.20 a	1.70 bt	1.10 a	1.30 d	1.60 d	2.80 d	0.90 d
		0.30 gl	0.90 a	0.80 d	0.20 l		0.20 g	0.70 a
		0.20 d	0.50 d	0.70 bt				0.70 bt
		0.20 bt	0.40 bl	0.30 u				0.10 bl
		0.10 vn	0.10 u	0.04 bl				0.10 u
		0.10 k	0.10 gl					0.10 gl
		0.02 bl						0.03 l
								0.01 vn
								0.01 k
								0.00 g

* Species > 0.1% are shown; <0.01% reported as 0.00. Values represent percentage volume by species. Letter codes denote tree species: a—*Quercus robur*, ar—*Quercus rubra*, b—*Betula pendula* and *Betula pubescens*, bl—*Salix caprea*, bt—*Alnus incana*, d—*Populus tremula*, e—*Picea abies*, ed—*Picea pungens*, g—*Ulmus glabra*, gl—*Salix fragilis* and *Salix alba*, j—*Alnus glutinosa*, k—*Acer platanoides*, ka—*Aesculus hippocastanum*, kk—other hardwoods, kp—*Pinus mugo*, ku—*Acer negundo*, l—*Tilia cordata*, m—*Larix* sp., p—*Pinus sylvestris*, pb—*Pinus banksiana*, s—*Ulmus minor*, sb—*Carpinus betulus*, t—*Populus* sp., u—*Fraxinus excelsior*, vk—*Robinia pseudoacacia*, and vn—*Ulmus laevis*.

**Table A3:** Age-class structure of tree growing stock by forest type-series. Forest type-series are grouped according to successional pathways, with three-phase trajectories dominated by colonizers (stand initiation, stem exclusion, steady state) and four-phase trajectories in which understory reinitiation produces mixed colonizer and successor assemblages.

Stand-Level Data	Stand Age Classes							LNFI 2024 Mean
	≤20	21–40	41–60	61–80	81–100	101–120	≥121	
Three-phase forest trajectory (all tree species): cl, v, vm, msp/s, cal/s, lsp/s, csp/s, c/s, cir/s								
Species composition by growing stock volume *	42.80 p	28.30 b	43.70 p	70.90 p	70.90 p	79.10 p	82.40 p	65.60 p
	30.90 j	27.50 p	24.90 j	9.80 e	9.80 e	14.00 e	13.00 e	11.40 e
	13.50 b	22.30 j	17.50 b	8.90 b	8.90 b	3.70 b	3.10 b	10.60 j
	5.50 e	13.20 e	10.60 e	8.80 j	8.80 j	2.60 j	0.80 j	10.20 b
	3.30 bt	5.30 d	1.50 d	0.80 d	0.80 d	0.30 a	0.60 a	1.10 d
	1.90 d	1.80 bt	1.00 bt	0.40 a	0.40 a	0.20 d	0.10 d	0.40 bt
	1.00 a	0.50 a	0.30 a	0.20 bt	0.20 bt	0.10 k	0.01 k	0.40 a
	0.60 bl	0.40 bl	0.20 gl	0.10 k	0.10 k	0.03 l	0.01 g	0.10 bl
	0.30 k	0.30 gl	0.10 l	0.03 l	0.03 l	0.01 bl		0.10 gl
	0.10 u	0.20 u	0.10 k	0.02 bl	0.02 bl	0.00 bt		0.10 k
	0.10 t	0.10 g	0.10 ar	0.02 u	0.02 u			0.05 l
	0.00 ar	0.10 pb	0.10 bl	0.02 gl	0.02 gl			0.03 u
		0.10 k	0.04 u	0.01 sb	0.01 sb			0.01 ar
		0.03 l	0.02 kp	0.01 ku	0.01 ku			0.01 g
		0.01 vn	0.01 g	0.00 pb	0.00 pb			0.01 pb
			0.00 pb	0.00 g	0.00 g			0.00 sb
			0.00 t					0.00 kp
								0.00 t
								0.00 ku
								0.00 vn
Four-phase forest trajectory (all tree species): m, ox, mox, fil/s, ur/s, hox, oxn, aeg, cmh								
Species composition by growing stock volume *	23.30 b	27.70 e	27.10 e	26.40 p	34.20 e	39.70 p	42.10 p	27.10 e
	17.60 d	22.30 b	19.20 b	25.00 e	28.50 p	29.20 e	24.60 e	21.30 p
	14.60 j	12.90 d	15.00 p	19.90 b	14.90 b	11.20 b	15.80 a	18.30 b
	13.20 e	12.60 bt	12.30 j	9.30 j	7.70 d	7.70 a	4.30 k	9.10 d
	9.00 bt	10.70 j	9.80 d	9.00 d	4.60 a	3.30 d	3.90 b	8.90 j
	8.50 a	5.30 p	9.70 bt	3.90 a	4.20 j	2.70 l	3.00 l	5.60 bt
	6.60 p	3.10 a	2.60 a	2.40 bt	1.90 l	2.20 j	2.20 d	4.50 a
	1.60 l	1.50 bl	1.20 k	1.90 l	1.20 k	1.40 k	1.20 j	1.50 l
	1.60 k	1.10 k	0.90 l	1.60 k	0.80 bt	1.00 sb	1.20 u	1.40 k
	1.50 bl	1.00 u	0.60 u	0.70 u	0.80 u	0.70 u	0.80 sb	0.80 u
	1.40 u	0.60 l	0.60 bl	0.20 sb	0.60 sb	0.20 g	0.40 g	0.50 bl
	0.40 vn	0.50 gl	0.30 gl	0.20 bl	0.30 g	0.20 bt	0.30 bt	0.30 sb
	0.40 gl	0.20 g	0.30 g	0.20 g	0.10 vn	0.20 vn	0.10 bl	0.20 g
	0.10 sb	0.10 sb	0.10 vn	0.10 vn	0.10 bl	0.10 bl	0.02 gl	0.20 gl
	0.10 g	0.10 vn	0.10 sb	0.10 s	0.05 s	0.10 s		0.10 vn
	0.01 s	0.10 t	0.10 t	0.10 gl	0.02 ku			0.04 s
	0.00 ku	0.03 ar	0.04 s	0.00 ar	0.01 gl			0.03 t
	0.00 ed	0.03 s	0.04 m	0.00 ku				0.01 m
		0.00 ku	0.02 ku					0.01 ku
			0.01 ka					0.01 ar
			0.01 vk					0.00 ka
			0.00 kk					0.00 kk

* Species > 0.1% are shown; <0.01% reported as 0.00. Values represent percentage volume by species. Letter codes denote tree species: a—*Quercus robur*, ar—*Quercus rubra*, b—*Betula pendula* and *Betula pubescens*, bl—*Salix caprea*, bt—*Alnus incana*, d—*Populus tremula*, e—*Picea abies*, ed—*Picea pungens*, g—*Ulmus glabra*, gl—*Salix fragilis* and *Salix alba*, j—*Alnus glutinosa*, k—*Acer platanoides*, kk—other hardwoods, kp—*Pinus mugo*, ku—*Acer negundo*, l—*Tilia cordata*, m—*Larix* sp., p—*Pinus sylvestris*, pb—*Pinus banksiana*, s—*Ulmus minor*, sb—*Carpinus betulus*, t—*Populus* sp., u—*Fraxinus excelsior*, vk—*Robinia pseudoacacia*, and vn—*Ulmus laevis*.
